# The Glass Transition Temperature of Heterogeneous Biopolymer Systems

**DOI:** 10.1021/acs.biomac.2c01356

**Published:** 2023-03-08

**Authors:** Suellen Pereira Espíndola, Ben Norder, Ger J. M. Koper, Stephen J. Picken

**Affiliations:** Advanced Soft Matter, Department of Chemical Engineering, Faculty of Applied Sciences, Delft University of Technology, Van der Maasweg 9, 2629 HZ Delft, The Netherlands

## Abstract

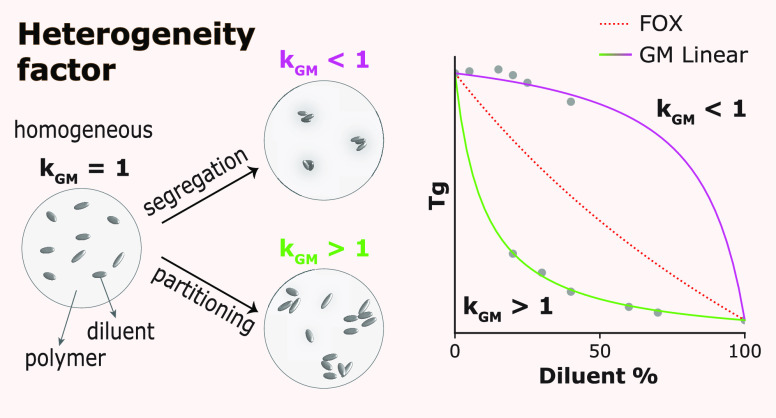

Biopolymers are abundant, renewable, and biodegradable resources. However, bio-based materials often require toughening additives, like (co)polymers or small plasticizing molecules. Plasticization is monitored via the glass transition temperature versus diluent content. To describe this, several thermodynamic models exist; nevertheless, most expressions are phenomenological and lead to over-parametrization. They also fail to describe the influence of sample history and the degree of miscibility via structure–property relationships. We propose a new model to deal with semi-compatible systems: the generalized mean model, which can classify diluent segregation or partitioning. When the constant *k*_GM_ is below unity, the addition of plasticizers has hardly any effect, and in some cases, even anti-plasticization is observed. On the other hand, when the *k*_GM_ is above unity, the system is highly plasticized even for a small addition of the plasticizer compound, which indicates that the plasticizer locally has a higher concentration. To showcase the model, we studied Na-alginate films with increasing sizes of sugar alcohols. Our *k*_GM_ analysis showed that blends have properties that depend on specific polymer interactions and morphological size effects. Finally, we also modeled other plasticized (bio)polymer systems from the literature, concluding that they all tend to have a heterogeneous nature.

## Introduction

Global concerns over climate change, plastic pollution, and scarcity of resources have made several industries actively look for alternative and sustainable material sources. Biopolymers are a great alternative with many applications already developed in food, agriculture, biomedical, and composite fields.^1−3^ However, solid-state materials consisting of polysaccharides and proteins are often too brittle and not workable.^4^ This is a classical materials design dilemma, where films are either (too) stiff and brittle or tough and (too) ductile. A common alternative for toughening polymeric materials is blending them with a diluent.^5−7^ This includes both (co)polymers and small non-volatile molecules, which can be added to decrease the polymer’s glass transition temperature (*Tg*). Therefore, further exploring our understanding of polymer-polymer and polymer–diluent systems is fundamental for developing improved biodegradable and sustainable biopolymer-based applications.

Biopolymer blends are frequently required because they can combine the specific properties of different materials in one. Often, small molecules are applied to plasticize the biopolymer, which provides better toughness and avoids catastrophic brittle failure. The ideal plasticizer for such biopolymer-based materials must be non-toxic, biodegradable, and preferably derived from natural sources.^4^ For instance, there are many reports of materials composed of polyols, oligosaccharides, citrates, lactates, vegetable oils, and tannins as natural additives.^8−10^ Even though the literature using bio-based materials and plasticizer agents is growing, their application is often investigated by trial and error. Furthermore, very few research studies exist on how to select a plasticizer, with most of them being phenomenological and case-specific. In addition, sample history, miscibility, and the extent of (local) phase separation are ignored. Therefore, how component compatibility affects the barrier, thermal, and mechanical properties of biopolymers is poorly addressed. As a side note, it is probably fair to say that living tissues, materials in nature with structural and mechanical functions, such as teeth, bones, wood, wool, and silk, obtain their sometimes excellent properties by virtue of components that bring about the required amount of mobility.

### Glass Transition Model Proposal

**Chart 1:**
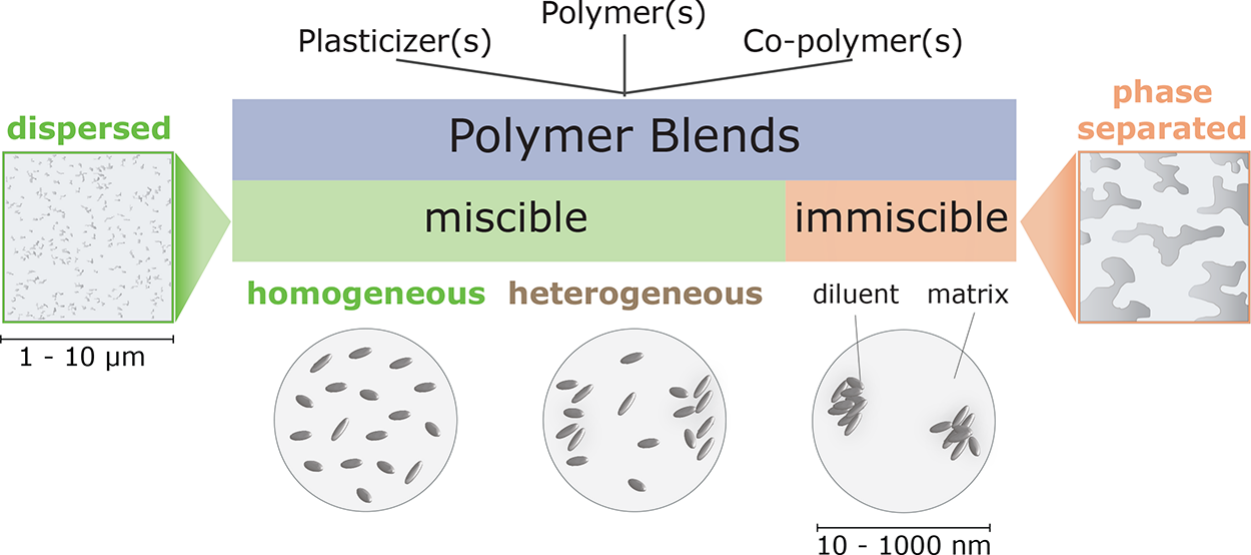
Morphology Classification of Polymer Systems We Propose for Glass Transition (*Tg*) Modeling Based on the Degree of Miscibilitya

Polymer blend miscibility is often studied via the determination of *Tg*, in which a single measured transition temperature identifies compatible systems.^11^ In truth, due to chain connectivity, even in miscible systems, the components will effectively experience distinct levels of mobility or relaxation times associated to a glass transition.^12^ For binary systems, several thermodynamical models for predicting the averaged *Tg* have been proposed, for instance, using the Gordon–Taylor and Couchman–Karasz expressions.^7,13−15^ In product engineering, the Fox equation^6^ is frequently applied, which also appears as a limiting form of the Couchman–Karasz expression. Nevertheless, it is seldom mentioned that these theories are only intended for the case of full compatibility. This is far from the general case of (bio)polymer mixtures, which may have a complex chemical composition and might show multiple conformations and variable levels of polydispersity. Due to this molecular complexity, the nanostructures that evolve from mixing biomacromolecules and diluents are expected to be locally heterogeneous in composition. On a supramolecular scale, there is local organization of mixed components, i.e., a certain level of segregation or partitioning may be recognized (Chart 1). Experimentally, the *Tg* of such heterogeneous blends is identified by one broad transition. The prediction of the *Tg,* even in binary systems, will also be difficult due to possible component interactions.^16^ As a result of concentration fluctuations^17^ and specific interactions, data can show negative or positive deviations from the usual rule of mixing for *Tg*.

a Images inside boxes and circles relate to the microscale and nanoscale, respectively.

Not surprisingly, there have been plenty of reports on how models based on the Couchman–Karasz expression fail to describe systems lacking true miscibility.^15,18,19^ Consequently, data from inhomogeneous mixtures are usually fitted by including additional correction terms to these equations. This leads to phenomenological expressions and over-parameterization. To work with semi-compatible systems, we propose an alternative working model, the generalized mean (linear) or *GM*(*L*) model. Within this framework, the conventional Fox equation becomes a particular case of our model, that of a homogeneous miscible system. It is based on the rather obvious idea that *Tg* is connected to interactions (enthalpy) and degrees of freedom (entropy) using a second-order phase transition-like framework, where the enthalpy and entropy type terms are averaged as a function of composition.

### Model Case Study with Alginate-Polyols

To showcase the use of the *GM*(*L*) model, we have performed a systematic study on the general plasticization effects of Na-Alginate with polyols. We have selected sugar alcohols as polyols because they provide a series of increasing H-bonded interactions and sizes. Then, we investigated if a single master curve of plasticization occurs based on a plasticizer’s mass fraction or molar density of the interacting functional group. Additionally, we used this system to investigate the interactions and the degree of miscibility based on our *Tg* modeling.

Finally, we demonstrate how the *GM*(*L*) model can be applied to an extensive list of *Tg* datasets from the literature. We explore the general understanding of static heterogeneity in (bio)polymer mixtures by evaluating the model’s constant *k*_GM_. Special attention is given to complex, multicomponent biopolymer systems since we believe the current theoretical background on *Tg* for this class of materials has yet to be adequately addressed.

## Experimental Section

### Materials

Sodium alginate (high ratio of mannuronic:guluronic acid, *M*_w_ ≈ 12–40 kDa), ethylene glycol, glycerol, meso-erythritol, d-(+)-arabitol, d-mannitol, and d-sorbitol were purchased from Sigma–Aldrich.

### Methods for Na-Alginate-(Sugar Alcohol) Films

#### Plasticization and Film Casting

The alginate-polyol films were prepared by the solution-casting method. Different sugar alcohols ((CHOH)_*n*_H_2_, where *n* is varying) with increasing chain length were tested to investigate the plasticizing effect on the developed films. Namely, ethylene glycol (C_2_), glycerol (C_3_), erythritol (C_4_), arabitol (C_5_), sorbitol (C_6_), and mannitol (C_6_) were used as plasticizers. The film preparation procedure is described as follows: a 5 wt % stock solution of Na-alginate in demineralized water was prepared. Afterward, an appropriate amount of dissolved plasticizer (5 wt %, in demineralized water) was added into separate film-forming solutions at a final dry mass of 0 to 50 wt % plasticizer (alginate basis, pH 8). The film-forming solutions with different plasticizer content were carefully homogenized with a glass rod, avoiding bubble formation until uniformly blended, and cast into polystyrene Petri dishes. The exact weight was calculated separately for each plasticizer to result in films with a thickness of about 0.15 mm. The freshly cast films were placed in ambient conditions (at 50 RH and RT), and different drying environments were tested. After around 3 to 5 days of drying, the free-standing films were peeled from the casting surfaces for analysis. Water is a natural plasticizer for hygroscopic alginate films, and sorption/desorption phenomena occur depending on the ambient conditions. Hence, to evaluate only the effects of polyol addition to the films, the cut film specimens were vacuum dried for 1 day at 40 °C and kept in a desiccator containing silica gel until immediately before analysis. For glycerol and sorbitol, the humid films (ambient, ∼50% RH) were also analyzed for comparison (Supporting Information, Figure S12 and Table S1).

#### Thermogravimetric Analysis

It was possible to add C_2_ to alginate and cast thin films. However, vacuum drying also removed part of this plasticizer from the matrix since it is too volatile. Hence, to measure the exact C_2_ content in the dried films, thermogravimetric analysis (TGA) was carried out on a PerkinElmer TGA 8000. The measurements were performed on samples of about 5 mg placed in a corundum crucible from 30–300 °C at a heating rate of 5 °C min^–1^ under a nitrogen atmosphere with an isothermal step at 90 °C for 30 min. TGA was also used for determining the water content in films of C_3_ and C_6_ equilibrated to ambient relative humidity using similar scans. TGA results can be found in the Supporting Information, Figure S5 and Table S1.

#### Dynamic Mechanical Thermal Analysis

Dynamic mechanical thermal analysis (DMTA) was performed on a PerkinElmer DMA-7e. DMTA experiments on the plasticized films were performed in tensile mode at a frequency of 1 Hz, a −100 to 180 °C temperature range, and a heat rate of 5 °C min^–1^ with film dimensions of roughly 20.0 mm × 3.0 mm × 0.1 mm. The thickness of the films was measured with the aid of a digital micrometer. The resulting glass transition was observed by the abrupt change in storage modulus slope and corresponding loss modulus maximum. This event is often called a polymer’s alpha relaxation. When possible, the *Tg* was estimated from duplicate measurements.

#### Glass Transition Modeling

To this experimental data, models were applied based on a *Tg* rule of mixing of polymer and plasticizer contributions. For convention, *Tg*_1_ and *Tg*_2_ are expressed as polymer and diluent components, respectively, with high and low *Tg* values. We chose to fit the often-applied Fox model:^6^
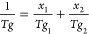
1where *x_i_* and *Tg_i_* denote the molar fraction and glass transition of components 1 and 2, respectively.

Further, we also fitted our model of interest, the generalized mean linear (*GM*(*L*)):
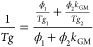
2where ϕ_*i*_ and *Tg_i_* denote the volume fraction and glass transition of components 1 and 2, respectively, and *k*_GM_ denotes the model constant. A full description of the Fox model and the *GM*(*L*) models we propose here can be found in Appendix A (Supporting Information). Curve fitting using nonlinear least squares was performed for all the studied plasticizers using a Python code and the function *scipy.optimise.curve_fit*, which employs a trust region reflective algorithm.^20^ The *Tg* values for alginate-polyol were optimized case by case but allowed to range from −200 to 200 °C. In addition, the *Tg* of alginate was not initially constrained to be the same in all systems. No initialization values were given. The goodness-of-fit of models was evaluated by the total sum of squares (TSS), *p*-value, and standard error of the regression *S*.

### Modeling (Bio)polymer Mixtures from Literature

Fox and *GM*(*L*) models, eqs 1 and 2, were also tested to an extended dataset of (bio)polymer blends carefully gathered from the literature. For completion, the full *GM* model was also investigated with alpha and beta values set to be positive (Supporting Information, Figure S15, and Table S3), as this constraint on the exponents is required to ensure convergence.

## Results and Discussion

### Blends of Na-Alginate-(Sugar Alcohol)

The glass transition temperature is a dynamic property with great current interest, since it is tied to thermal, mechanical, and vibrational properties. For instance, it is used not only to determine operational and processing temperature ranges but also influences mechanical properties like stiffness, tensile strength, toughness, hardness, and impact resistance.^4,5^ Besides standard thermomechanical factors, it has been related to materials’ adhesive and healing mechanisms.^21−23^ Indirectly, *Tg* will also affect electrical, optical, diffusion, and barrier properties, physical aging, and environmental stability. The *Tg* of polymers can conventionally be assessed through thermal analysis, i.e., evaluating the dependence of a specific volume, jump in heat capacity, or change in modulus on temperature.

**Figure 1:**
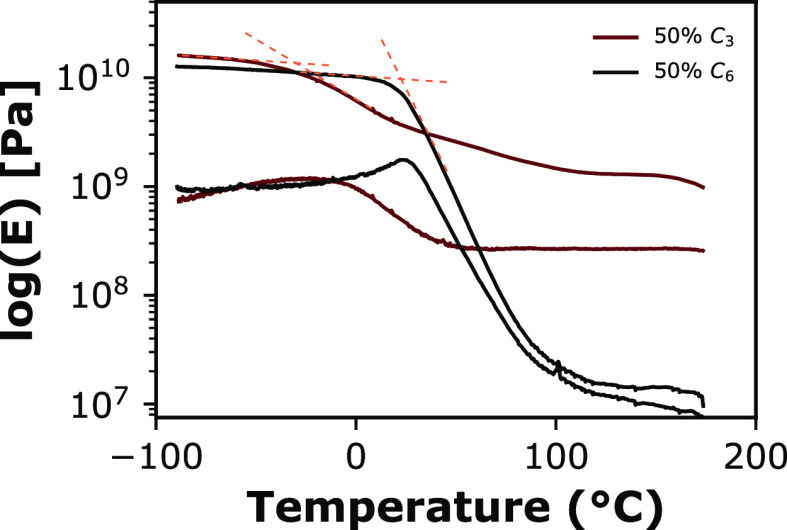
DMTA analysis of Na-alginate-(sugar alcohol) films containing 50 wt % glycerol (C_3_) or sorbitol (C_6_). The higher and lower curves respectively show the temperature dependence of the storage (*E*′) and loss (*E*″) moduli. The dashed lines demonstrate how to estimate the temperature of a glass transition relaxation on a logarithmic modulus scale.

We chose to study the *Tg* of alginate-polyols by DMTA due to the high sensitivity of this method to polymer relaxations. The onset of the primary relaxation (alpha) corresponds to the mobility of the main chain, which happens only at *Tg*. Figure 1 shows examples of alpha relaxation identification from highly plasticized alginate films. In a polymer blend, the presence of one major relaxation is a sign of miscibility. DMTA showed primarily one main relaxation for dried samples, while samples left to ambient relative humidity resulted in the appearance of additional relaxations. This phenomenon is further explained in Supporting Information (Figures S6 to S12). After the *Tg* event, the magnitude of the rubber plateau in alginate blends varied with the type and size of added sugar alcohol.

**Figure 2:**
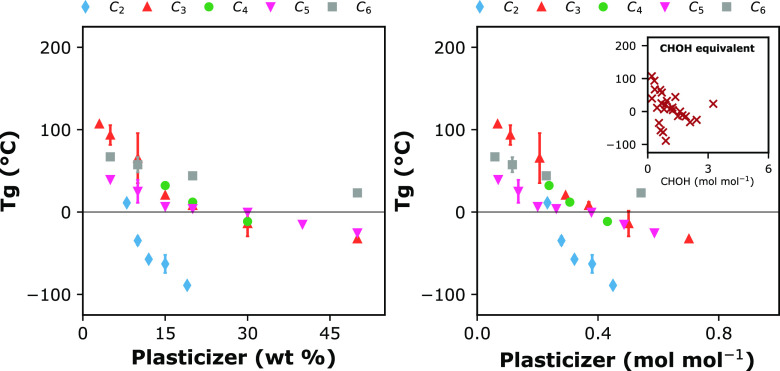
Glass transition temperature (*Tg*) of mixtures of Na-alginate and sugar alcohol plasticizers in mass (A) or molar (B) fractions. The sugar alcohols are depicted from C_2_ to C_6_, based on the general formula (CHOH)_n_H_2_. Inset: *Tg* over plasticizer fractions translated to CHOH molar equivalents. Error bars indicate standard deviation.

In Figure 2, the obtained *Tg* values of dry blends are plotted against the diluent’s mass or equivalent molar fractions. Irrespective of the way the plasticizer content is evaluated, one cannot find a master curve of *Tg* versus plasticizer. This is surprising since sugar alcohols have the same generic chemical formula: (CHOH)_*n*_H_2_. Even if we reduce all polyol concentrations to [CHOH] equivalents, as shown in the inset graph of Figure 2B, there is no obvious universal *Tg* pattern. This strong argument supports that even though they are miscible, the sugar alcohols do not interact in the same way with the alginate chains. Similar findings of a plasticizer-dependent interaction and compatibility limit have previously been reported for the systems of alginate and C_3_ and C_6_ polyols.^24,25^

**Figure 3:**
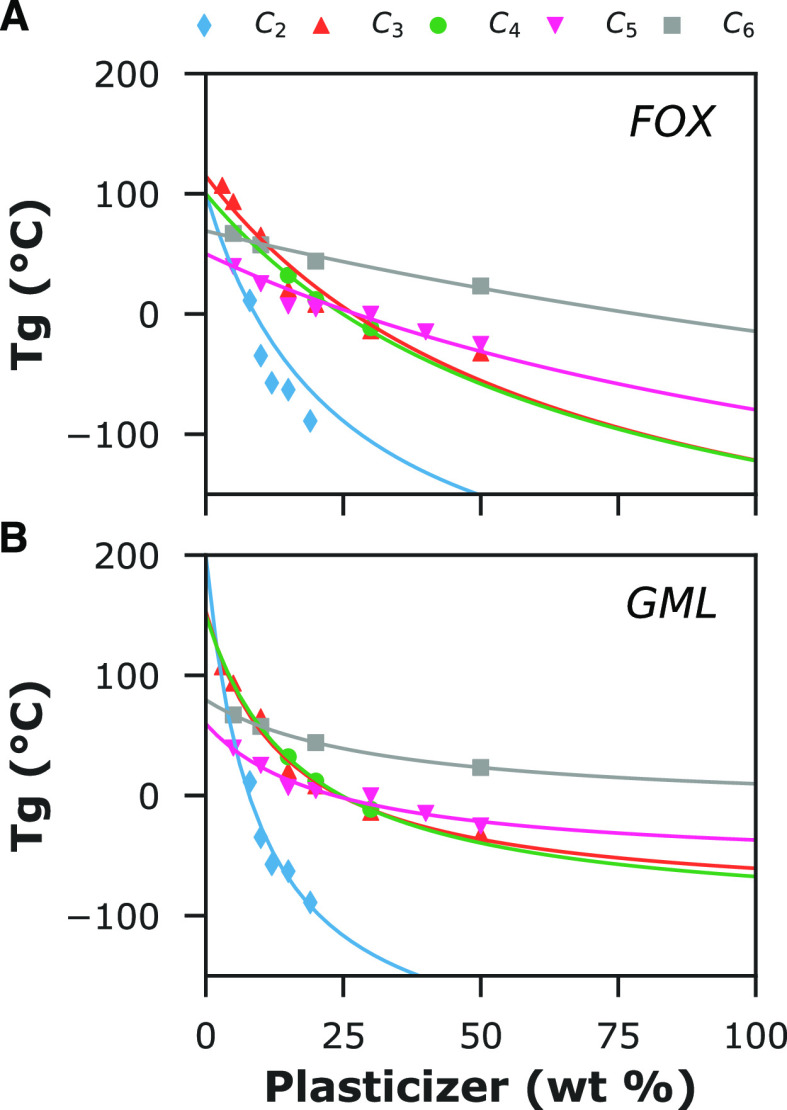
Experimental and calculated values of glass transition temperature (*Tg*) for Na-alginate-(sugar alcohols). (A) Fox model (FOX); (B) generalized mean linear model (*GML*). Curve-fitting was allowed using appropriate boundary values for the individual *Tg* parameters.

We have performed model curve fitting to evaluate this *Tg* data (Figure 3). The widely applied Fox model is a simple harmonic mean of *Tg* contributions. We observe that this model does not fit the data of most sugar alcohol mixtures well. Even though the data fit for C_3_ and C_5_ are statistically accurate (*p* < 0.05, *S* < 31 °C), the values of *Tg* are poorly predicted (Table 1). Alternatively, the linearization of the generalized mean model, *GML*, shows an excellent prediction of datasets (*p* < 0.01, *S* < 15 °C). In addition, the *GML* goodness-of-fit is equivalent to that obtained via the analogous Gordon–Taylor equation. However, when possible, the *Tg* of individual components should also be experimentally obtained prior to model fitting. Hence, Table 1 values are to be taken as a likelihood of *Tg* within the mixture. We also note that setting appropriate boundary conditions for the individual *Tg* contributions was necessary for a good-quality *GML* fit.

**Table 1:** Glass Transition Parameters and Statistics Obtained from Curve-Fitting Fox or Generalized Mean Linear (*GML*) Models to Na-Alginate-(Sugar Alcohol) Datasets^a^

polyol	model	*Tg*_1_ (°C)	*Tg*_2_ (°C)	model constant, *k* (fit ± st. error)	TSS	*p* value	*S* (°C)
C_2_	Fox	100*	–200*	1	7781	0.0173	31.27
	*GML*	200*	–196	1.93 ± 8.08	5665	0.0071	14.64
C_3_	Fox	115	–122	1	17,436	0.0006	16.30
	*GML*	153	–61	3.92 ± 1.31	17,432	0.0002	8.31
C_4_	Fox	100*	–122	1	954	0.0374	4.25
	*GML*	150*	–67	3.30 ± N/A	954	N/A	N/A
C_5_	Fox	50*	–80	1	2944	0.0009	8.10
	*GML*	60	–37	3.75 ± 2.01	2944	0.0008	5.19
C_6_	Fox	69	–14	1	1077	0.0252	4.12
	*GML*	79	10	3.32 ± 0.00	1077	1.96 × 10^–14^	6.62× 10^–13^

a TSS: total sum of squares from the regression model; S: standard error of regression coefficient; N/A: not applicable due to zero degree of freedom. *Values corresponded to the used boundaries for the parameter.

**Figure 4:**
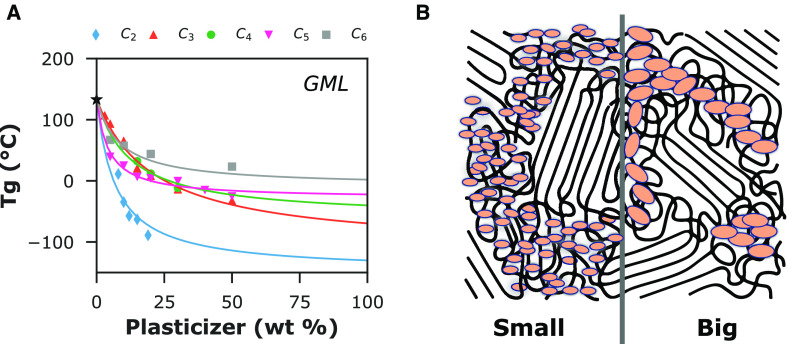
(A) Experimental and calculated values of glass transition temperature (*Tg*) for Na-alginate-(sugar alcohols). The generalized mean linear (*GML*) model was fitted by assuming a fixed glassy polymer *Tg* (star) and appropriate boundary values for the plasticizer *Tg*. (B) Illustration showing the difference in steric partitioning of a small or big plasticizer in a semi-crystalline polymer.

Another advantage of the *Tg* models is that they can be used to indirectly determine the (virtual) transition of a glassy polymer by extrapolation to zero diluent concentration. Like many biopolymers,^26^ the *Tg* of pristine Na-alginate cannot be determined since thermal decomposition is observed before the transition. From Figure 3, all alginate-polyol datasets point toward a virtual Na-alginate *Tg* or *Tg*_1_, between 60 to 180 °C. Russo et al.^27^ have previously reported a *Tg* of 133 °C for Na-alginate based on differential scanning calorimetry of relatively dry specimens. This seems like a reasonable estimate of M-rich alginate with the remaining tightly bound water. We can use this as a unified value for the Na-alginate-(sugar alcohol) data and retrofit it to the *GML* model (Figure 4). The approximated value of 133 °C does seem to fit well with most datasets except for C_6_ (*p* > 0.05). That can be explained by the fact that the estimated *Tg* of Na-alginate is not an actual material property but an apparent one, with a plasticizer changing the internal structure. For C_5_ and C_6_ polyols, both studied models result in a lower *Tg* estimated for the neat alginate. It might be that the addition of a larger H-bonding plasticizer partially disrupted the semi-crystallinity of this block-copolymer. Evidence of semi-crystallinity was also found in powder XRD, haziness of alginate-polyol films, and a high modulus rubber plateau in the DMTA results (Supporting Information, Figures S1, S4, and S6–S11). Additionally, the plasticizer itself also showed a tendency to crystallize, as in the case of films with high content of C_4_ or another C_6_ compound (mannitol) (Supporting Information, Figures S2–S4).

Nevertheless, the new *GML* fits described all datasets well as a physical model (*S* < 25 °C) (Table 2). The fit values obtained for polyol *Tg* were in good agreement with the literature (Supporting Information, Table S2). The retrofit allows us to appropriately compare the alginate *Tg* over the increasing plasticizer fraction. For small-size polyols, C_2_ and C_3_, the *Tg* of the blend decreases rapidly with more polyol until a plateau is reached. For larger polyols, C_5_ and C_6_, we observe a *Tg* plateau already at a relatively lower polyol fraction. The *GML* model introduces a new constant, *k*_GM_, which can be interpreted to arise from the static partitioning of the polymer/plasticizer fractions. From Table 2, the positive *k*_GM_ values for all Na-alginate-(sugar alcohols) indicate a substantial deviation from the case of miscibility (*k*_GM_ = 1, analogous to the Fox model). This *k*_GM_ value of >1 means that the effective plasticizer concentration appears to be higher than expected, resulting in a lower *Tg* at lower plasticizer content due to partitioning. The most significant deviations were observed for C_5_ and C_6_ alcohols, which might be explained by their size, causing substantial steric interaction-driven partitioning in a semi-crystalline matrix. We envision that a smaller plasticizer, such as C_2_ polyol, would better penetrate and fill the free volume of the amorphous domains in contrast to C_5_ and C_6_ (Figure 4B), which would not be able to penetrate into the amorphous phase adjacent to the crystalline regions. In fact, it should be noticed that a certain level of semi-crystallinity will always result in *k*_GM_ > 1 as the concentration of diluent in the amorphous matrix regions will effectively be higher than expected from the overall composition because the crystalline regions are not (or much less) available.

**Table 2:** Glass Transition Parameters and Statistics Obtained after Curve Fitting Generalized Mean Linear (GML) Model on Na-Alginate-(Sugar Alcohol) Datasets Using a Unified *Tg* for the Glassy Polymer^a^

polyol	*Tg*_1_ (°C)	*Tg*_2_ (°C)	boundaries *Tg*_2_ (°C)	*k*_GM_ (fit ± st. error)	TSS	*p* value	*S* (°C)
C_2_	133*	–130	–100 ± 30	5.37 ± 42.85	5675	0.0432	24.96
C_3_	133*	–70	–79 ± 30	2.83 ± 1.23	17,439	0.0005	10.06
C_4_	133*	–40	–10 ± 30	5.19 ± N/A	956	N/A	N/A
C_5_	133*	–22	–22 ± 30	14.90 ± 14.65	2945	0.0003	7.89
C_6_	133*	2	–28 ± 30	8.71 ± 20.26	1109	0.2949	20.84

a TSS: total sum of squares from the regression model; S: standard error of regression coefficient; N/A: not applicable due to zero degree of freedom; * Assumed value for neat Na-alginate as found by Russo and co-workers.^27^

The actual extent of semi-crystalline/amorphous fractions and domain sizes is experimentally challenging to be obtained. In theory, it might have been resolved by X-ray scattering and analysis of the crystalline peak width using the Scherrer equation. For the case of alginate semi-crystallinity, the measured crystalline degree would also serve to estimate the amorphous phase that is available for the plasticizer. Thus, this amorphous space would be equivalent to the length of heterogeneity. This rationale obviously assumes the plasticizer to be amorphous in the resulting mixture. Nevertheless, getting good information on this from scattering techniques is very challenging and would constitute an entire additional study. One advantage of the *GML* model is that the quantification of any crystalline or immobile fraction is not necessary to fit *Tg* data over composition and identify levels of heterogeneity, therefore, it might serve to better determine heterogeneity from structural analysis as it provides an expectation value.

Indeed, only the *GML* model resulted in good descriptive curves for the *Tg* of alginate-(sugar alcohols). We believe that this can only be due to partial miscibility or, to put it in another way, local heterogeneity of polymer and plasticizer distribution in such blends. In some cases, the components’ specific interactions might cause this phenomenon, i.e., semi-crystallinity, H-bonding, and chirality. In fact, H-bonds are well known to affect the final semi-crystallinity of polymers.^28−30^ Another influence might be the chirality of C_4_ to C_6_ sugar alcohols, causing preferential sites in the plasticizer distribution. In general, the case of alginate blends is a good example that specific interaction contributions and partitioning need to be considered. The Fox model neglects such interactions as it is based solely on the entropic contributions of components in a fully miscible blend. However, fully and partially miscible blends can be described with *GML*, where the constant *k*_GM_ acts as a factor representing the heterogeneity. Regarding thermodynamics, *k*_GM_ can also be interpreted as a convoluted factor of both (second order) enthalpic and entropic contributions.

### Heterogeneity Constant

**Chart 2:**
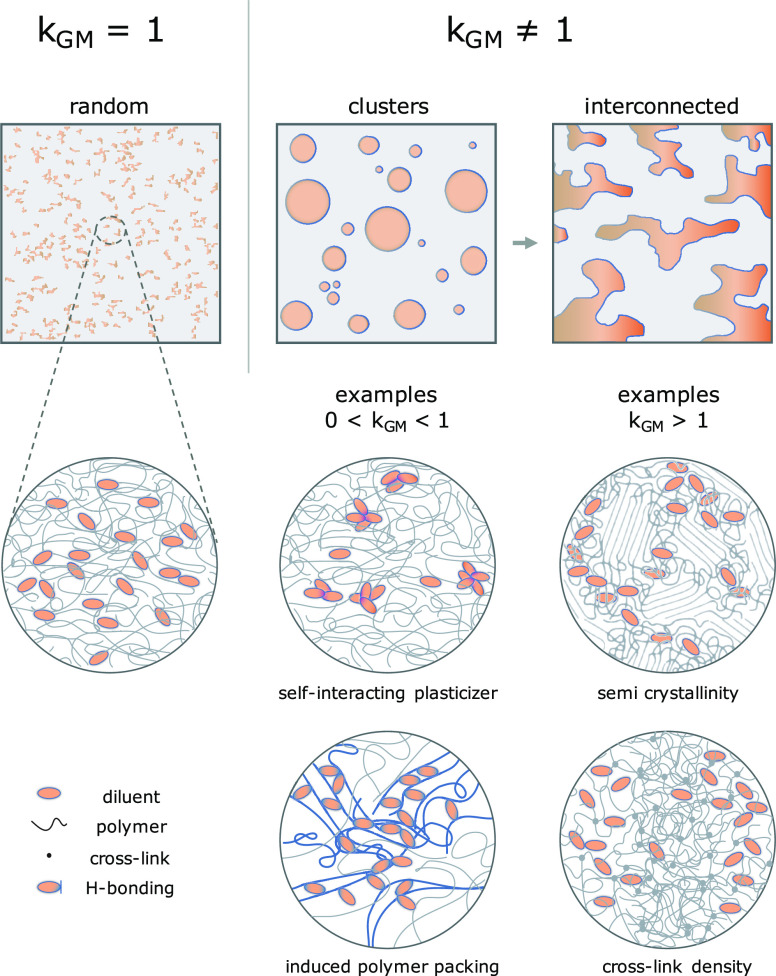
Illustrations of a Homogeneously Distributed (*k*_GM_ = 1) or Partitioned Diluent (*k*_GM_ ≠ 1) in a Glassy Polymer Hosta

Other situations can cause static heterogeneity (*k*_GM_ ≠ 1) of a diluent distribution within a glassy polymer matrix. A link to the *Tg* property can easily be interpreted if coupled with the free volume theory. For comparison purposes, one could classify heterogeneous blends resulting in *k*_GM_ below or above unity, as illustrated in Chart 2. Both semi-crystallinity and crosslinking density variations can result in *Tg* values lower than predicted by the Fox model (*k*_GM_ > 1). In the first case, as shown here with alginate-(polyols), steric partitioning effects can happen if the glassy matrix forms some crystalline or densely packed domains. With regards to cross-linking, a densely packed polymer–polymer network can also effectively create irregular boundaries to plasticizer clusters,^7^ even though cross-linking generically increases *Tg*, the plasticizer would be at a higher concentration in the available regions (Chart 2).

a Boxes: microscale; circles: nanoscale.

A few examples, usually in a low diluent content regime, can lead to *Tg* values higher than predicted by the Fox model (0 < *k*_GM_ < 1). For instance, often at lower volume fractions, a plasticizer with a tendency for segregation can result in the free volume cavities of the polymer being filled with plasticizer, e.g., the anti-plasticization effect. It can also happen that interactions between the diluent/plasticizer and stiff polymer chains will enhance the level of polymer packing.^31^ This is analogous to the anti-solvent polymer packing effect.^32^

In both cases, further diluent clustering will frequently lead to microscopic phase separation of a blend, which is often observed in the form of opacity and coarsening of the phase-separated structure. Further, the diluent phase will either exudate out of the solid matrix, forming a binary or ternary-phased system, or even locally crystallize, thereby removing the plasticizer from the polymer matrix entirely. These two events are macroscopic. From a thermodynamic perspective, the *k*_GM_ factor can also characterize blends from miscible to immiscible states as it deviates away from unity.

It is interesting to note that the proposed local heterogeneity is not necessarily an undesirable phenomenon on a micro- or nanoscopic level. Heterogeneous plasticization will create zones of local plasticization and lubrication of amorphous polymer chains, resulting in mobility, pliability, and increased toughness. Simultaneously, the material’s structural integrity or tenacity is still provided by the regions of low plasticizer content, preventing creep and flow. In other words, good plasticizers should not be too compatible with the polymer. It should be enough to mobilize without solubilizing the whole system.

Within the context of binary polymer blends, it might be helpful to consider the self-concentration approach proposed by Lodge and McLeish (2000) for blends with large *Tg_i_* difference.^12^ The theory states that the average composition of the local environment around a certain component must be enriched by itself because of chain connectivity. Therefore, even for homogeneous blends, each polymer will effectively experience its own composition dependent dynamics and effective *Tg*. This unavoidable segregation happens at the level of the chain Kuhn length and can get further exacerbated by the abovementioned specific interactions and clustering/segregation phenomena. The *k*_GM_ constant is obtained assuming a mixture’s single or averaged *Tg*, where the product *k*_GM_ϕ_2_ is an estimation of the (anti)plasticizer phase. Therefore, *k*_GM_ is also a convoluted expression of local heterogeneity from multiple length scales: at chain segment and cluster levels. A theoretical relationship between the effective self-concentrations of a polymer/diluent in a blend and the *GM*(*L*) model constant is still lacking, which shall be considered in future work.

### General Application to Heterogeneous Mixtures

In this section, we demonstrate the versatility of the *GM*(*L*) model for polymer blends of synthetic and biological origin. The goal is to show via partitioning factor *k*_GM_ how easily systems fall outside true miscibility and simple rule-of-mixing theory, especially when biopolymers are used. These peculiar states of miscibility can arise from strong specific molecular interactions, steric effects, and conformational changes (morphology), depending strongly on sample history. It also makes sense to present this data compilation to connect our current work to general plasticization and anti-plasticization phenomena.

**Figure 5:**
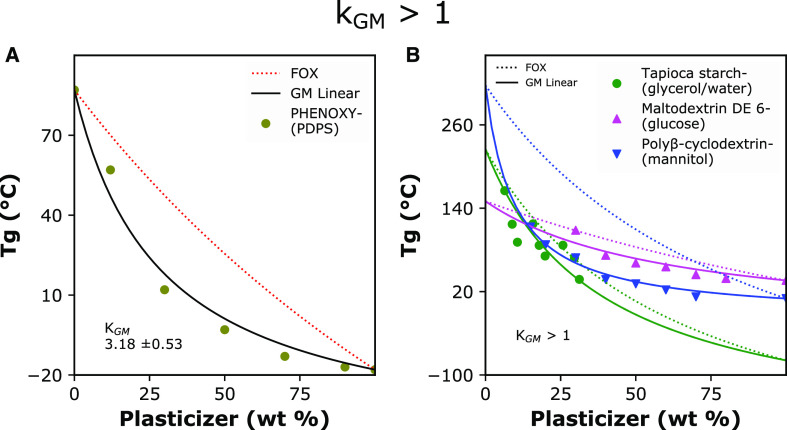
Experimental and calculated values of glass transition temperature (*Tg*) for several datasets displaying a greater-than-expected decrease with a plasticizer (or *k*_GM_ > 1). (A) Synthetic polymer blend of the polyhydroxyether of bisphenol A (PHENOXY) and poly(2,2-dimethyl-1,3-propylene succinate) (PDPS). (B) Biopolymer-(plasticizer) mixtures. FOX: Fox model (dotted lines); GM Linear: generalized mean linear model (solid lines). Curve-fitting was performed using fixed values for the individual *Tg* parameters for demonstrative purposes. Data from Schneider, 1997;^31^ Chang, 2006;^33^ Linnenkugel et al., 2021;^15^ and Tabary et al., 2016.^37^

Figure 5 displays datasets with a greater decrease of *Tg* with diluent content (*k*_GM_ > 1) in contrast to what was predicted by Fox’s theory. We can interpret those results with the main rationale that the effective diluent volumetric fraction in a polymer matrix is higher than initially expected. In Figure 5A, the synthetic polymer blend of Phenoxy resin or poly(hydroxy ether of bisphenol-A), with an aliphatic polyester of succinate (PDPS), shows decreased *Tg* values in comparison with those predicted by entropic contributions (Fox theory).^31^ It is common knowledge in polymer processing that a physical blending of polymers needs strong specific interactions to result in miscibility. In this case, H-bonding between carbonyl groups of succinate ester and hydroxyl of Phenoxy should overcome intramolecular cohesion and favor miscibility. However, the *Tg* curve gives us additional information that there must be competing energetic interactions since *k*_GM_ > 1. We speculate that this makes sense, since while Phenoxy is a blocky and amphiphilic polymer, the succinate polyester is polar, which might cause a more loosely packed structure, thus affecting the free volume of the blend.

The plasticized mixture of tapioca starch-(glycerol/water) also follows a *k*_GM_ > 1 trend (Figure 5). The difference between Fox and *GML* curves is subtle, as seen in Table 3. However, this becomes relevant if we know that the films formed a semi-crystalline matrix upon drying,^33^ possibly resulting in plasticizer partitioning. In starches, the recrystallization of amylose and sometimes amylopectin can fundamentally influence properties, e.g., water vapor permeability and toughness. Water from moisture can further change *Tg* considerably.^34,35^ Consequently, the effect of water in crystalline biopolymer blends should always be evaluated or excluded.

**Table 3:** Glass Transition Parameters and Statistics Obtained from Curve Fitting Fox and Generalized Mean Linear (*GM*(*L*)) Model for Datasets Showing Deviations from the Rule of Mixing^a^

system	*Tg*_1_ (°C)	*Tg*_2_ (°C)	model	model constant, *k* (fit ± st. error)	TSS	*p* value	*S* (°C)	references
Phenoxy–(PDPS)	87*	–18*	Fox	1	11,167	N/A	18.92	Schneider, 1997^31^
			*GML*	3.18 ± 0.53	10,183	2.52 × 10^–6^	5.31	
tapioca starch–(glycerol/water)	225* *	–79* *	Fox	1	15,759	N/A	28.57	Chang, 2006^33^
			*GML*	1.41 ± 0.19	10,615	0.0009	24.24	
maltodextrin DE 6–(glucose)	150* *	36*	Fox	1	5107	N/A	13.07	Linnenkugel et al., 2021^15^
			*GML*	1.91 ± 0.72	3779	0.0024	9.44	
poly-cyclodextrin–(mannitol)	317*	10*	Fox	1	61,190	N/A	67.79	Tabary et al., 2016^37^
			*GML*	5.60 ± 0.57	9984	6.95 × 10^–7^	5.56	
PBIAz–(ULTEM)	427*	217*	Fox	1	112,615	N/A	30.08	Schneider 1997^31^
			*GML*	0.38 ± 0.04	98,908	6.04 × 10^–13^	12.88	
sugar palm starch–(glycerol)	238*	–79* *	Fox	1	41,787	N/A	96.51	Sahari et al., 2013^38^
			*GML*	0.14 ± 0.01	2526	0.0005	4.96	
chitosan–(polyols)	125*	–79* *	Fox	1	8196	N/A	54.83	Ma et al., 2019^40^
			*GML*	0.09 ± 48.92	1124	0.7109	18.82	
corn starch/chitosan–(glycerol/water)	90* *	–79* *	Fox	1	10,514	N/A	52.92	Liu et al., 2013^45^
			*GML*	0.11 ± 0.19	250	0.3069	11.35	

a *Values used to fit models were extracted from the original data source; **Values used to fit models were absent and, thus, estimated by this study for illustrative purposes.

Continuing on Figure 5B, the system of maltodextrin-glucose^15^ is interesting because the polymer is a polydisperse derivative of starch. The maltodextrin analyzed did not show crystallite sites; nevertheless, again, we find that *k*_GM_ > 1. This can maybe be explained by the strong H-bonding interactions between glucose and maltodextrin, creating plasticizer–polymer and polymer–polymer domains with possible steric effects.^15^ One could consider evaluating the extent of blending steric effects by systematically increasing the plasticizer size, for instance, from smaller polyols to different-chain length polyethylene glycols.^36^

Complexes of guest–host chemistry and cross-linking will also impact *Tg* values and trends. In Figure 5B, a blend between a methylated polymer of β-cyclodextrin (polyβ-cyclodextrin) and mannitol^37^ shows a significant divergence from Fox’s prediction. The polymer cyclodextrin can form hydrophobic-core inclusion complexes for drug delivery. The degree of di-ester cross-linking of the polymer with citrate is also essential to this application and *Tg* determination. The 36 wt % cross-linked and plasticized material was produced via a melting process. We could relate the *Tg* divergence from the ideal rule of mixing theory to the increased free volume of heterogeneous cross-linking of the sample, influencing molecular packing, along with mannitol’s resistance to filling in the hydrophilic core of cyclodextrin inclusions.

**Figure 6:**
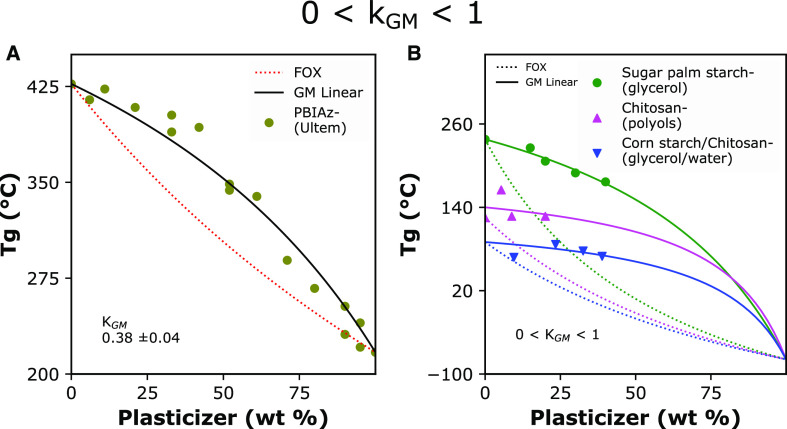
Experimental and calculated values of glass transition temperature (*Tg*) for several datasets displaying lower than expected decrease with a plasticizer (or 0 < *k*_GM_ < 1). (A) Synthetic polymer blend of polybenzimidazole (PBIAz) and Ultem polyetherimide (Ultem). (B) Biopolymer–(plasticizer) mixtures. FOX: Fox model (dotted lines); GM Linear: generalized mean linear model (solid lines). Curve-fitting was performed using fixed values for the individual *Tg* parameters for demonstration purposes. Data from Schneider 1997;^31^ Sahari et al., 2013;^38^ Ma et al., 2019;^40^ and Liu et al., 2013.^45^

A good plasticizer will result in pliable and durable materials that are easy to process by increasing the mixture’s free volume or lowering the *Tg*. In turn, the mixed material should be tougher than the initial glassy polymer. In Figure 6, datasets show an increase of *Tg* with polymer or plasticizer content. This increase deviates completely from the Fox theory; for *GM*(*L*), it represents the case of 0 < *k*_GM_ < 1 (Table 3). In fact, the synthetic blend of polybenzimidazole with commercial polyetherimide (Ultem)^31^ resulted in anti-plasticization. This phenomenon happens when we observe an increase in overall specific density with diluent addition.^5^ In extreme cases, even phase separation can occur. Within the partial miscibility region, anti-plasticization can be desired if a material’s performance needs to be improved. However, a slowly decreasing *Tg* or even a plateau with increasing diluent content often seems counterintuitive. Polybenzimidazoles (PBIAz) are high-performance engineering thermoplastics with very stiff aromatic polymer cores and high *Tg* values (> 400 °C). In Figure 6A, we propose that the strong H-bonding interaction of PBIAz with Ultem via amine groups may cause additional stiffness via chain confinement. Hence, in this case, the favorable interactions between the chains cause the *Tg* to hardly decrease, giving rise to anti-plasticization.

For sugar palm starch-(glycerol) plasticized films (data in Figure 6B), strong H-bonding interactions between glycerol and amylose/amylopectin resulted in high *Tg* values.^38,39^ In particular, for starches, processing can be crucial. Starch samples often must be gelatinized at high temperatures (>130 °C) to obtain thermoplastic behavior. This could result in the plasticizer affecting the formation of crystalline domains from starch moieties. Similarly, systems of chitosan-polyols can also show significantly different properties depending on the strength of H-bonding interaction with the polymer backbone and overall moisture content (Figure 6). The chitosan data is shown with respect to the hydroxyl groups of sugar alcohols.^40^ It is important to note that with further diluent addition, aging, or the increment of water from moisture, systems can dramatically move from the anti-plasticized to the plasticized regime.^41−43^ This can be observed both in *Tg* and in mechanical performance. The plasticization shift will depend on how strong the energetic interactions (enthalpy-driven) are and how favorable the increase of free volume (entropy-driven) is. In polymer blends and plasticized systems with large Δ*Tg_i_*, this has been early on reported as a break (or cusp) in *Tg*-composition curves.^11,44^ The phenomena have mostly been attributed to a critical temperature, where a change in (strong) specific interactions with diluent fraction is observed.

Trends in multicomponent systems can also be interpreted similarly via *k*_GM_. A 1:1 starch/chitosan blend was produced by microfluidization.^45^ This blend was plasticized by glycerol and water, showing high anti-plasticization (0 < *k*_GM_ < 1) through strong H-bonding between the plasticizer and macromolecules. Previous works have only presented explicit thermodynamic solutions for multicomponent systems of polysaccharides, polyols, and water^8,15^ by working with Flory Huggins’s free volume theory and extending the Couchman–Karasz expression for *Tg*.

Locally heterogeneous mixtures are ubiquitous in materials based on (bio)polymers, showing complex thermodynamic behavior. We have observed that *k*_GM_ can be a helpful tool to investigate (bio)polymer-diluent miscibility and possibly derive insights into structure–property relationships. We note that the linearized *GML* model is intended only for systems in a continuum, i.e., with no phase separation, crystallization, or phase inversion. Alternatively, the original *GM* model can adopt complex shapes (Supporting Information, Figure S15). Yet, we do not recommend modeling immiscible systems instead of splitting the developed phases. Overall, this study is another example that the topic of glass transition is a complicated part of polymer science.^46^ For example, it is often very challenging to determine the *Tg* of neat and biopolymer mixtures because the thermal transitions are found above degradation, increasing with the strength of electrostatic interactions, cross-links, and branching. Nowadays, this topic has become even more relevant with the rapid pursuit of tailored biodegradable and sustainable materials.

## Summary and Conclusions

The generalized mean linear model, *GML*, works as a versatile model for studying the glass transition of polymer blends and plasticized systems. The model can be seen as a natural extension of the widely used Fox model (1956). In *GML*, if the constant *k*_GM_ is not 1, the system is not fully homogeneous—or Fox-like—and there is obvious evidence of heterogeneity or local demixing on a nanoscale. This can be explored via systematic studies to reveal the structure–property relationships of blends and elect a suitable and stable plasticizer for a specific application. To deal with strong interactions and heterogeneity, previous models have been proposed and modified, like Couchman–Karasz equations. However, the adopted solutions are often case-specific, phenomenological, and lead to over-parametrization, thus failing to describe the overall picture of (partial) miscibility.

This study showcases our *GML* model applied to predict the *Tg* of Na-alginate and polyols as plasticizing molecules. The experimental data on *Tg* clearly does not follow the Fox equation, while only the *GML* model can fit the results. This indicates that heterogeneity is important in alginate-polyol, as is also substantiated by the observed size effect of the type of polyol on *Tg* curves. This proposed heterogeneity indeed becomes apparent at higher plasticizer content from the overall sample appearance and via microscopy. Hence, sample processing history also becomes important. This heterogeneous plasticizer distribution is presumably caused by regio-specific interactions in the alginate-polyol system, such as the semi-crystallinity of the polymer matrix and steric effects in amorphous domains, as is apparent from our results. In addition, the *GML* model can easily describe the heterogeneity present in a wide range of diverse (bio)polymer blends, demonstrating its utility in analyzing complex polymer materials and even anti-plasticization phenomena.

Based on the above and considering the heterogeneous nature of biopolymers, research on bio-based systems can benefit from the *GML* approach. Living organisms produce biopolymers that are designed to be complex in structure and interactions, containing chiral macromolecular arrangements taking the form of helices and sheets or even showing semi-crystallinity. The already-present structural heterogeneity is often amplified by extraction and (re)processing conditions. In the case of solvent-based processes, the scale of heterogeneity can be large, especially if elevated temperatures are used. The chemical structure, therefore, is not so well-controlled. In addition, electrostatic interactions are nearly always present, which adds additional specific interactions not customarily found in fossil-based polymers. In summary, one could say that the molecular morphology of materials based on biopolymers and natural plasticizers is intrinsically heterogeneous. Hence, such systems should nearly always fall outside the commonly used rules of mixing for the thermal properties of polymer blends.

## Supporting Information Available

Appendix A (derivation generalized mean model); Experimental design; powder XRD of Na-Alginates; photographs of thin films of alginate-polyol, also under different drying conditions; optical microscopy of thin films of alginate-polyol; TGA curves of alginate-polyols for C_2_ plasticizer final concentration; DMTA curves of alginate-polyols under dry and humid conditions used for obtaining *Tg*; residual plot for Fox and *GM*(*L*) models for alginate-polyols; sugar alcohol *Tg*; *GM*(*L*) model applied to complex datasets from literature (PDF)

## ABBREVIATIONS

C_2_: ethylene glycol
C_3_: glycerol
C_4_: erythritol
C_5_: arabitol
C_6_: sorbitol
TGA: thermogravimetric analysis
DMTA: dynamic mechanical thermal analysis
*GM*: generalized mean model
*GML*: generalized mean linear model
*k_GM_*: constant of the generalized mean linear model
