# Supplementary material for: The Glass Transition Temperature of Heterogeneous Biopolymer Systems
Source: Biomacromolecules. 2023 Mar 8;24(4):1627–37. doi: 10.1021/acs.biomac.2c01356 (PMC10091355; doi:10.1021/acs.biomac.2c01356)
Supplement: Supplementary file 1 — bm2c01356_si_001.pdf [file bm2c01356_si_001.pdf]

SUPPORTING INFORMATION

The glass transition temperature of heterogeneous biopolymer systems

Suellen Pereira Espíndola, Ben Norder, Ger Koper, Stephen J. Picken**

Advanced Soft Matter, Department of Chemical Engineering, Faculty of Applied Sciences, Delft University of Technology, Van der Maasweg 9, 2629 HZ Delft, The Netherlands

*Correspondence to:

Suellen Pereira Espindola (E-mail: S.PereiraEspindola-1@tudelft.nl)

Stephen J. Picken (E-mail: S.J.Picken@tudelft.nl)

Contents

APPENDIX A 3

Glass Transition Modelling 3

Generalised Mean Model 5

Summary of Thermodynamic Relations 7

Experimental design 9

Photos of Na-alginate films dried at different conditions: haziness & XRD of semi-crystalline Na-alginate film 10

Photos of Na-alginate-(polyol) films 12

Photos of Na-alginate-(C_4) films dried under ambient and N_2 flow conditions (suppressed polyol recrystallization) 14

Optical microscopy of Na-alginate-(polyol) films 15

TGA of Na-Alginate-(C_2) films to determine plasticizer content 16

DMTA of Na-alginate-(polyol) films 17

DMTA and TGA of Na-alginate, C_3, and C_6 polyol films equilibrated to ambient moisture 21

Residuals plot over independent variable for Fox and GML models for alginate-polyols 23

Sugar alcohol glass transition temperature 25

Full GM model applied to datasets from literature 26

References 29

APPENDIX A

In this section, we give a short summary of the glass transition theory of mixtures and show how the Generalised Mean model can be derived from a quasi-second order thermodynamic transition.

Glass Transition Modelling

Couchman and Karasz (1978) demonstrated an interesting model for the effect of the composition of binary mixtures on *Tg*[1]. The model was successful for compatible polymer-polymers blends and also polymer-diluent systems[2,3]. The model derivation was based on writing that *Tg* is not a first-order thermodynamic transition; more specifically, on the property of continuity of a system's specific entropy at *Tg*. Hence, the integration of Δc_p of a polymer blend results in the following simplified expression:

$$x_1 \int_{Tg_1}^{Tg} (c_{p1}^{l} - c_{p1}^{g}) d \ln T + x_2 \int_{Tg_2}^{Tg} (c_{p2}^{l} - c_{p2}^{g}) d \ln T + \Delta_{mix}\mathrm{S} = 0 \qquad \text{(S1)}$$

where *x*, *Tg*, c_p, *l*, *g* denote molar (or volume) fraction, glass transition, specific heat, supercooled liquid and glassy states of pure components 1 and 2, respectively. Originally, the authors were also careful to mention that the commonly neglected entropic terms just above and below the transition ($\Delta_{mix}\mathrm{S} = \Delta S_{mix}^{l} - \Delta S_{mix}^{g}$), should be included if there are excess entropy changes upon mixing (conformational, thermal, etc.). From the perspective of Gibbs free energy, it is true that an enthalpic relationship of eq S1 also holds. Irrespective of the form (entropy or volume continuity conditions), note that for most systems the c_p property of components undergoes a finite discontinuity at the transition.

All models derived from eq S1 have the activation or mobility of chain units as a fundamental principle, corrected through Δc_p values. Another ubiquitous *Tg* model is the expression earlier

proposed by Gordon and Taylor (1953)[4], which can also be rearranged into another simplification of eq S1. To derive this relationship, we must rewrite $\Delta c_{p2}/\Delta c_{p1}$ into a constant, k_{GT}, so that:

$$Tg = \frac{x_1 Tg_1 + k_{\mathrm{GT}} x_2 Tg_2}{x_1 + k_{\mathrm{GT}} x_2} \qquad \text{(S2)}$$

In fact, the thermodynamic parameter k_{GT} is related to a constant coefficient of expansion (volume) during the transition. However, this phenomenological solution was conceived for ideally mixed copolymers. Thus, a simplification ($k_{GT} = 1$) results in a linear averaging of the *Tg,* which to our knowledge never occurs.

The most frequently used equation for the change in *Tg* is the phenomenological Fox (1956) expression[5–8]:

$$\frac{1}{Tg} = \frac{x_1}{Tg_1} + \frac{x_2}{Tg_2} \qquad \text{(S3)}$$

From equation (S1), it assumes that $\Delta c_{p2} * Tg_1/\Delta c_{p1}$ is a constant. This way, the predicted *Tg* becomes a simple rule of mixing of the entropic contributions of the pure components. Hence, simply put, it assumes no effect from enthalpic interactions upon mixing.

However, when we analyse *Tg* changes with an x_2 component, a large portion of experimental results show large deviations from those ideal mixture predictions. The most used forms of the discussed models either neglect excess thermodynamic property from mixing or make it elusive to work around such values since Δc_p corrections are not straightforward to interpret. Several other models have been proposed to tackle this, such as the models of Kwei (1984)[9], additional terms expanding Couchman-Karasz (eq 3)[3,10–12], and models based on virial coefficients[13]. Yet most of these approaches can easily result in over-parameterisation, and outcomes are hard to interpret.

Generalised Mean Model

Inspired by the simple harmonic mean expression proposed by Fox, we have developed a new working model for the *Tg* property in blends. Similar to previous studies, we assume *Tg* is a quasi-second-order transition according to the Ehrenfest classification. Thus, it can be written as $T_g = \frac{\Delta_s \partial H}{\Delta_s \partial S}$, where ∂H and ∂S are, respectively, second or *n*th-term partial derivatives of enthalpy and entropy at transition. If we further expand on this equality, we can take the relation in thermodynamic properties to be weighted in the form of a generalised or power mean instead of the Fox-like harmonic mean. It follows that the Generalised Mean (GM) model becomes:

$$Tg = \frac{\Delta_s \partial H}{\Delta_s \partial S} = \frac{(\phi_1 \Delta_s \partial {H_1}^{\beta} + \phi_2 \Delta_s \partial {H_2}^{\beta})^{1/\beta}}{(\phi_1 \Delta_s \partial {S_1}^{\alpha} + \phi_2 \Delta_s \partial {S_2}^{\alpha})^{1/\alpha}} \qquad \text{(S4)}$$

where ϕ_i,, α, β are volume or mass fraction of components 1 or 2, entropy exponential, and enthalpy exponential, respectively.

The expression S4 can be further arranged if we couple some of the partial derivative terms into a constant, k_{GM}. For convenience, k_{GM} is obtained by substituting the ratio of partial derivative enthalpies ∂H of components ($\Delta_s \partial H_i$). However, a similar output could have been obtained by using ∂S. Considering that the Tg of each component is $\Delta_s \partial H_i / \Delta_s \partial S_i$ and that k_{GM} can be expressed as $\Delta_s \partial H_2 / \Delta_s \partial H_1$, the full GM equation can be manipulated into the applicable form:

$$Tg = \frac{(\phi_1 {Tg_1}^{\beta} + \phi_2 (Tg_1 k_{GM})^{\beta})^{1/\beta}}{(\phi_1 + \phi_2 (\frac{Tg_1 k_{GM}}{Tg_2})^{\alpha})^{1/\alpha}} \qquad \text{(S4a)}$$

where ϕ_i, Tg_i, k_{GM}, α, β are volume or mass fraction of components 1 or 2, glass transition of components 1 or 2, model constant, entropy exponential, and enthalpy exponential, respectively. This model has five degrees of freedom and can resolve into nontrivial parabola or S-shaped curves by tuning the α and β exponents. Previously, such S-shaped data have been previously modelled using virial *Tg* models, e.g., for tetramethyl bisphenol-A polycarbonate-(polystyrene) blend[13].

Nevertheless, we noticed that most data take a simple form, and we can do a linearisation (α, β = 1). Hence, the linearised Generalised Mean model (*GML*) becomes:

$$\frac{1}{Tg} = \frac{\frac{\phi_1}{Tg_1} + \frac{\phi_2 k_{GM}}{Tg_2}}{\phi_1 + \phi_2 k_{GM}} \qquad \text{(S5)}$$

From this form, the model can also revert to the Fox equation (eq S3) if k_{GM} is 1, which explains our choice for defining k_{GM} from the enthalpy ratio. The constant k_{GM} can also be interpreted as a partitioning factor correcting the volume fraction of diluent (ϕ_2). Hence, it is a static measure of system partitioning or heterogeneity. The *GML* version resolves most nonlinear cases and is mathematically analogous to the Gordon-Taylor model (appears from assuming a ratio of partial derivative entropies ∂S). Moreover, *GML* is useful because excess property and possible structural changes can be easily monitored with one factor, k_{GM}.

The values and morphological states implied from fitting the *GM(L)* model are heavily influenced by sample history. Hence, drying and cooling rates will likely influence the assessed diluent partitioning. In particular, the cooling rate effect on sample history should be accounted for, since quenching can suppress the difference in *Tg*(s). It is also worth underlining that sensible experimental kinetic rates should be used for determining the thermal transition. The experiment observation times should obviously be probing the relaxation times of system components. Furthermore, the *Tg* property is known to broaden and increase logarithmically with the quench rate[14]. Although the time-dependent effects are not explored, the model is able to fit accordingly the *Tg* curves over composition, irrespective of the studied fabrication method or experimental rates employed.

Reliable *Tg* measurements are crucial for the findings of *GML* model fit to be valid. This should be ensured by selecting a sensitive enough technique, for instance DMTA, dielectric spectroscopy and modulated differential scanning calorimetry. These methods are less affected by broadening

effects at transition[15]. Yet Tg variations among methodologies as high as 20 °C are normally expected. In addition, adequate machine calibration and experimental conditions (environment, rate, strain, oscillatory parameters) need to be explored. Lastly, the data analysis step should be well reported for there are multiple standard ways to obtain *Tg*.

Summary of Thermodynamic Relations

The Gibbs energy *G(T, P, {n})* is a continuous function of its natural variables temperature *T*, pressure *P*, and composition, *{n}*, and has as derivatives over temperature entropy $S = -\frac{\partial G}{\partial T}$ and enthalpy $H = -T^2 \frac{\partial G/T}{\partial T}$. In the Ehrenfest sense, at a first-order transition, the Gibbs function is continuously differentiable everywhere except at the phase transition temperature, where the slope changes, so that *S* and *H* have jump-values, Δ*S* and Δ*H,* respectively. The jump values differ by a factor equal to the transition temperature, i.e.: $T_t = \frac{\Delta_t H}{\Delta_t S}$, where the subscript *t* denotes that the values are to be taken at the transition.

The entropy and enthalpy are continuous, except at first-order phase transitions, and their derivatives with temperature are related to the specific heat as: $\frac{\partial S}{\partial T} = \frac{c_p}{T}$ and $\frac{\partial H}{\partial T} = c_p$.

At a second order transition, the enthalpy and entropy are continuously differentiable everywhere except at the phase transition, where the slope changes so that the heat capacity has a jump value such that: $T_s = \frac{\Delta_s \frac{\partial H}{\partial T}}{\Delta_s \frac{\partial S}{\partial T}}$, where the subscript *s* denotes that the values are to be taken at the secondary transition. If a material's glass transition is assumed to be a (pseudo) second order phase transition, the same relation should apply to the jump-values of ∂*H* and ∂*S*. Nevertheless, the relationships mentioned above do not work for systems with c_p close to infinity at transition, i.e., lambda transitions, as is the case of systems with order-disorder evolution.

The blending of components will also involve thermodynamic mixing functions, i.e., the entropy of mixing $\Delta_{mix}S$ and enthalpy of mixing $\Delta_{mix}H$. For ideal miscible mixtures, the enthalpy of mixing is zero, and the entropy of mixing is positive from increased disorder. We can imagine entropy as the main driving force arising from the dispersion of components. Real mixtures often contain excess interaction of any of the pairs of components. This specific interaction(s) might result in enthalpy changes or additional entropy upon mixing, for instance, from molecular clustering. Depending on the magnitude of the enthalpy step from energetic interactions or adverse entropy, the total Gibbs energy becomes positive, and the system phase separates spontaneously. This would cause miscibility up to a certain composition (partial miscibility) or full immiscibly in a polymer blend. Excess functions can be calculated as the difference between $\Delta_{mix}S$ (or $\Delta_{mix}H$) of real and ideal solutions to investigate nonideal cases.

Experimental design

Chart S1. Experimental design used in fabrication of Na-alginate-(sugar alcohol) films

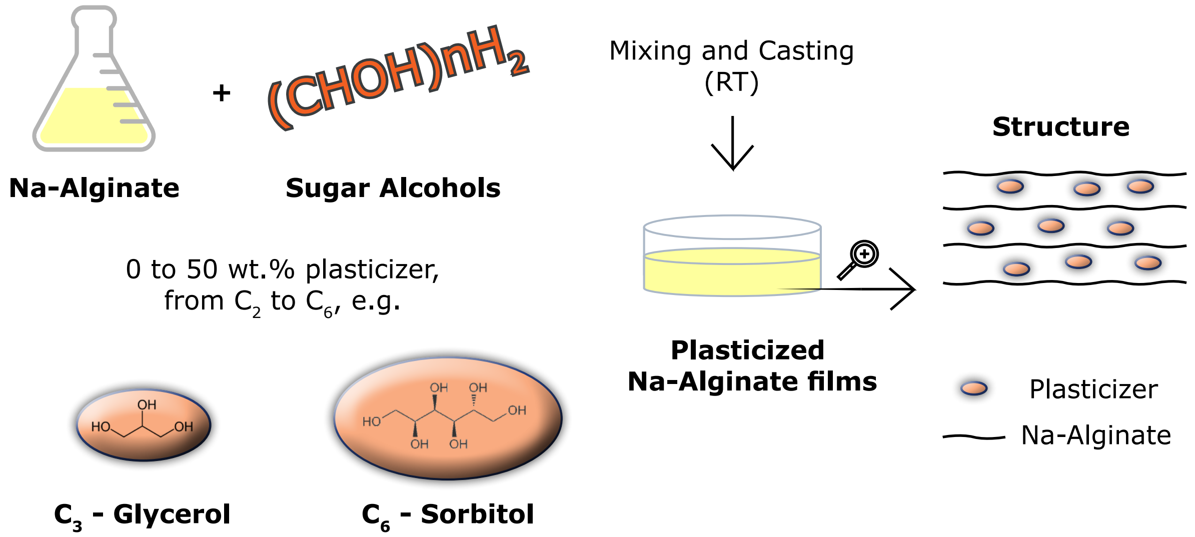

Photos of Na-alginate films dried at different conditions: haziness & XRD of semi-crystalline Na-alginate film

Rigid semi-crystalline structures can form in alginate as it is composed of mannuronate and guluronate building blocks. The visual aspect of alginate films can be linked to degree of this semi-crystallinity and, in blends, also to the degree of miscibility. The samples studied actually showed different degree of haziness depending on the rate of drying and type of sugar-alcohol used (Figure S1A). Haziness appeared specially when high fractions of polyol where present and at low drying rate (in a laminar air flow hood). Therefore, we have used this observation to screen which films were best uniformly blended and should be used in our study. Additionally, by analysing our Na-Alginate polymer with x-ray diffraction (XRD), we confirmed that the semi-crystalline structure is formed in solvent-casted films (2θ peaks between 10° and 20°, Figure S1B) in contrast to flash-dried samples, for instance, with freeze drying.

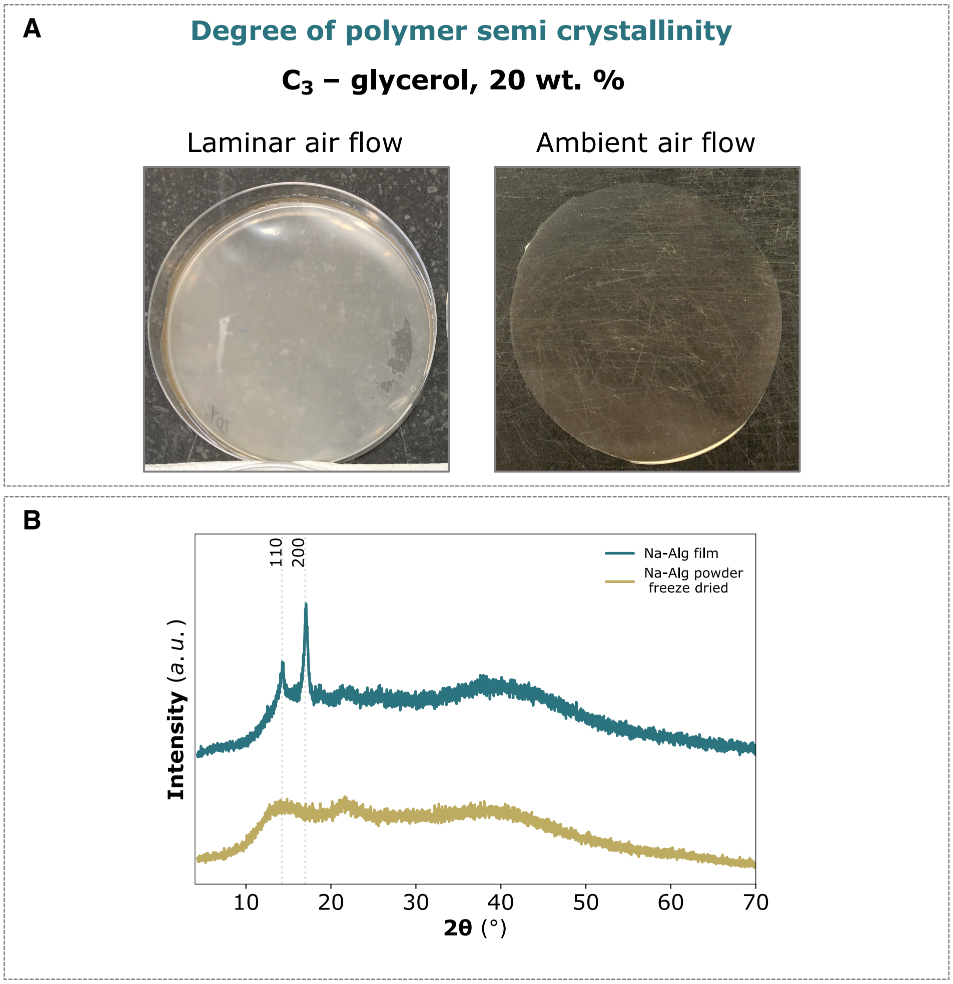

Figure S1. A) Photographs of Na-alginate-(20 wt.% glycerol) films casted under a weak laminar air flow (left) and laboratory ambient air flow (right). The left film is opaque and less homogeneous in contrast to right. B) X-ray diffraction of neat Na-alginate in the form of a slowly-dried film (top curve) and freeze-dried Na-alginate powder (bottom curve). The vertical lines indicate peaks of guluronic (110) and mannuronic acid (200) crystallite fractions. The lack of Bragg peaks for the freeze-dried sample indicates amorphous domains are obtained by using faster drying rates.

Photos of Na-alginate-(polyol) films

Free-standing films could be successfully produced by solvent-casting up to a plasticizer's compatibility limit. Under ambient drying conditions, it was possible to fabricate homogeneous transparent alginate films containing up to 50 wt.% plasticizer for most polyols (**Figure S2**). These films were pliable at ambient conditions from 15 wt.% polyol content. C_4 polyol had a high tendency for recrystallization and fast drying of the film under vigorous N_2 flow was required (**Figure S3**). Films could only be produced up to a 30 wt.% C_4 threshold. Hence, we can assume film drying conditions and storing played a role in the resulting properties. If such conditions are not handled properly, partial de-mixing, exudation and/or crystallization can occur. This compatibility limit is actually dependent both on specific interactions and sample processing (e.g., mixing and drying methods). For alginate-polyol, unwanted macroscopic effects such as crystallization and plasticizer migration had to be prevented, respectively, by fast evaporation and storing the films in a dry chamber. In fact, we have observed with optical microscopy that polyols showed a tendency to partially mix or crystallize at different concentrations (**Figure S4**). The polyol mannitol crystallized at lower concentrations than sorbitol, even though these compounds are isomers. Therefore, there were plenty signs of different internal structures depending on type and amount of diluent.

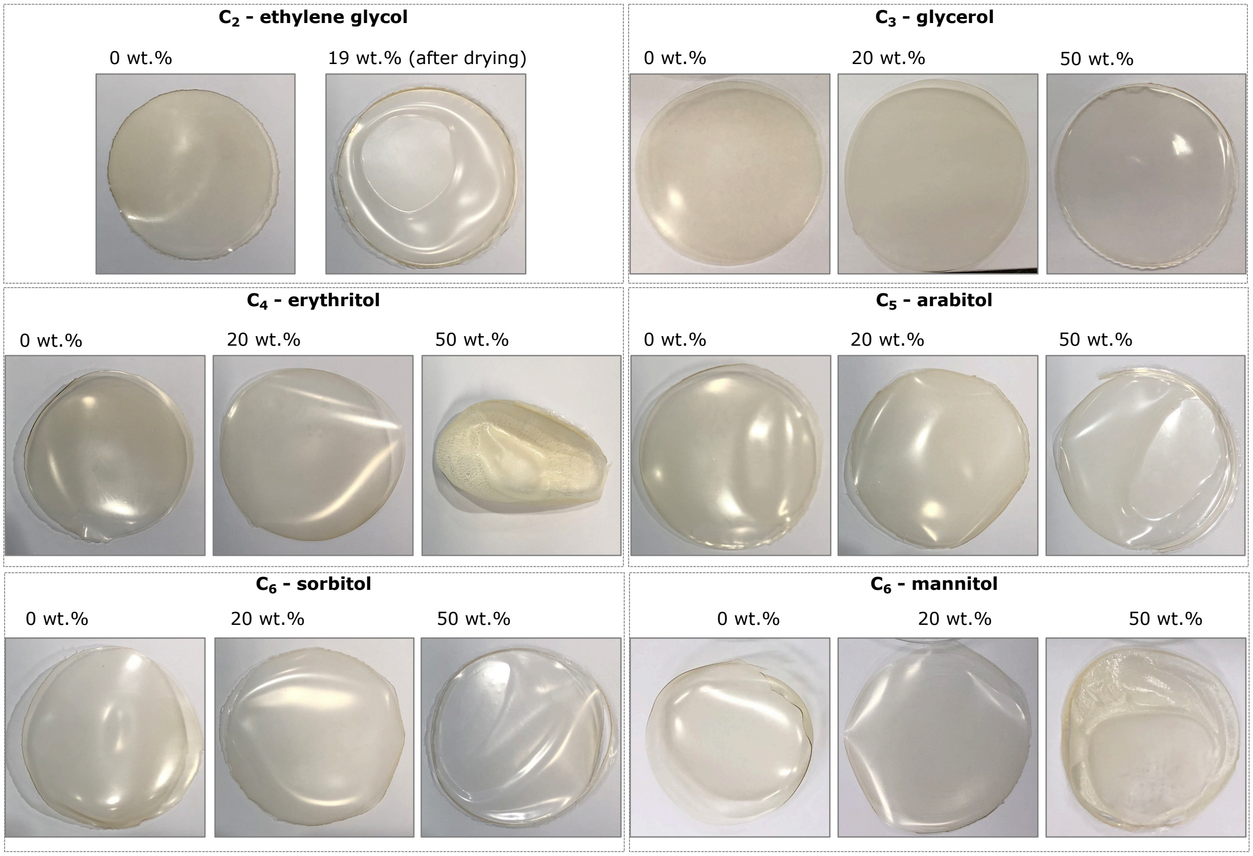

Figure S2. Photographs of Na-alginate-(sugar alcohol) films ranging from C_2 to C_6 type of plasticizer and containing 0, 20 and 50 wt.% fractions. C_2 could only be produced up to 19 wt.%, since vacuum drying removed some of its initial mass.

Photos of Na-alginate-(C_4) films dried under ambient and N_2 flow conditions (suppressed polyol recrystallization)

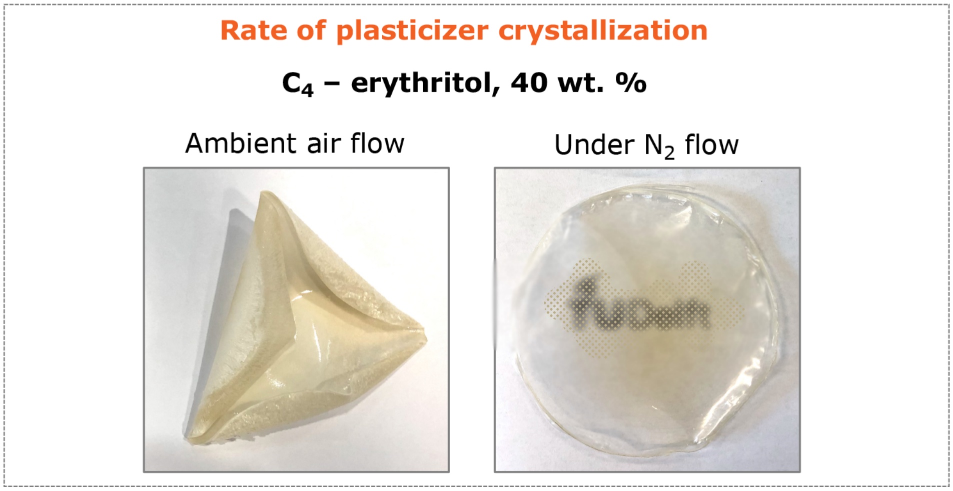

Figure S3. Photographs of Na-alginate-(40 wt.% erythritol) films casted under laboratory ambient air flow (left) and under vigorous N_2 flow (right) for a rapid and extensive drying. The right sample did not show signs of macroscopic C_4 recrystallization.

Optical microscopy of Na-alginate-(polyol) films

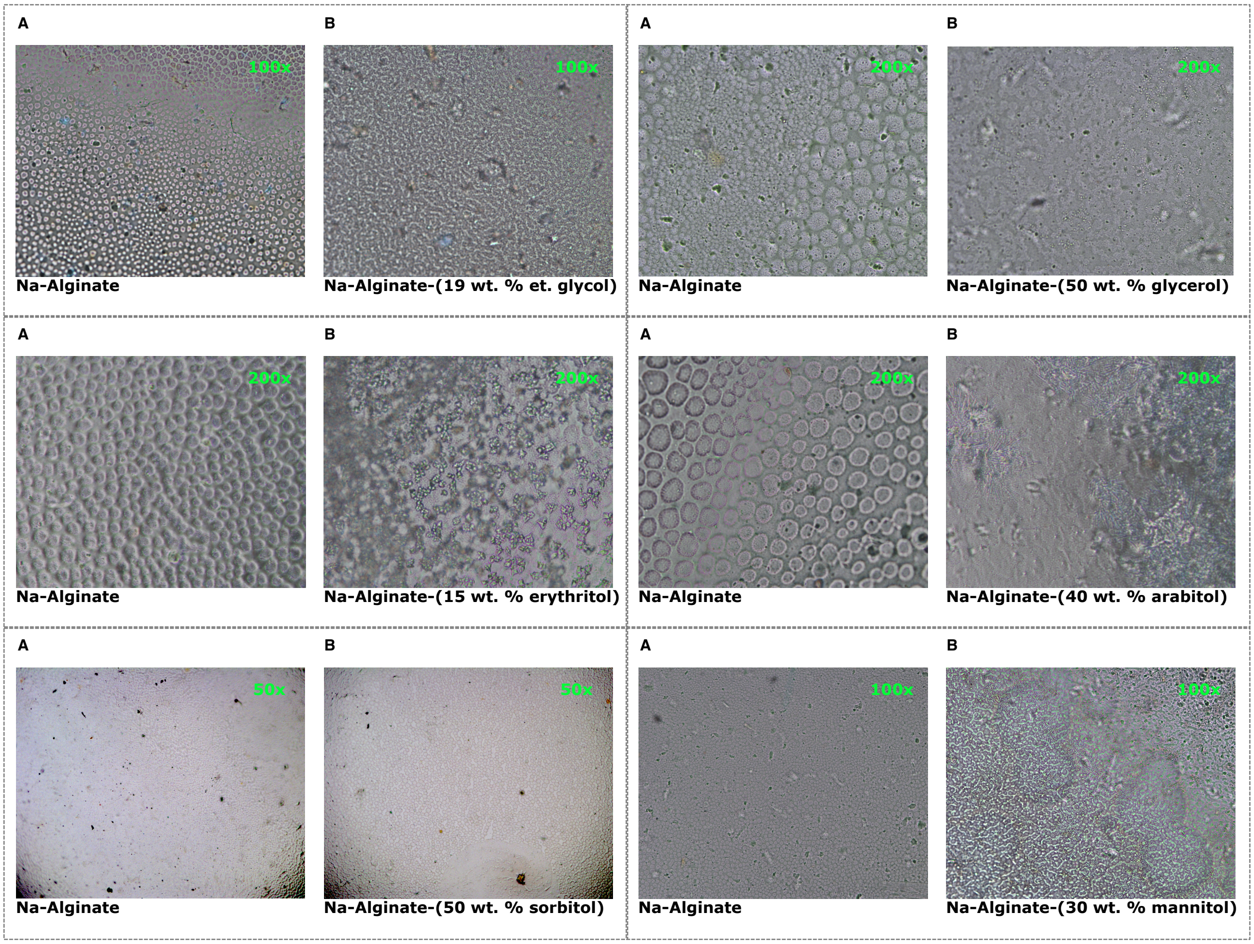

Figure S4. Optical microscopy of Na-alginate-(sugar alcohol) films. Images were taken of freshly casted samples after ambient exposure (~50 RH). Films of erythritol, arabitol and mannitol show clear signs of crystallization phenomena if dried at ambient conditions.

TGA of Na-Alginate-(C_2) films to determine plasticizer content

The remaining content of C_2 polyol was determined by subtracting weight loss values for pristine Na-Alginate from the ones obtained for the blends of Na-Alginate-(C_2). The concentrations found are shown in legend of Figure S5 as wt.%.

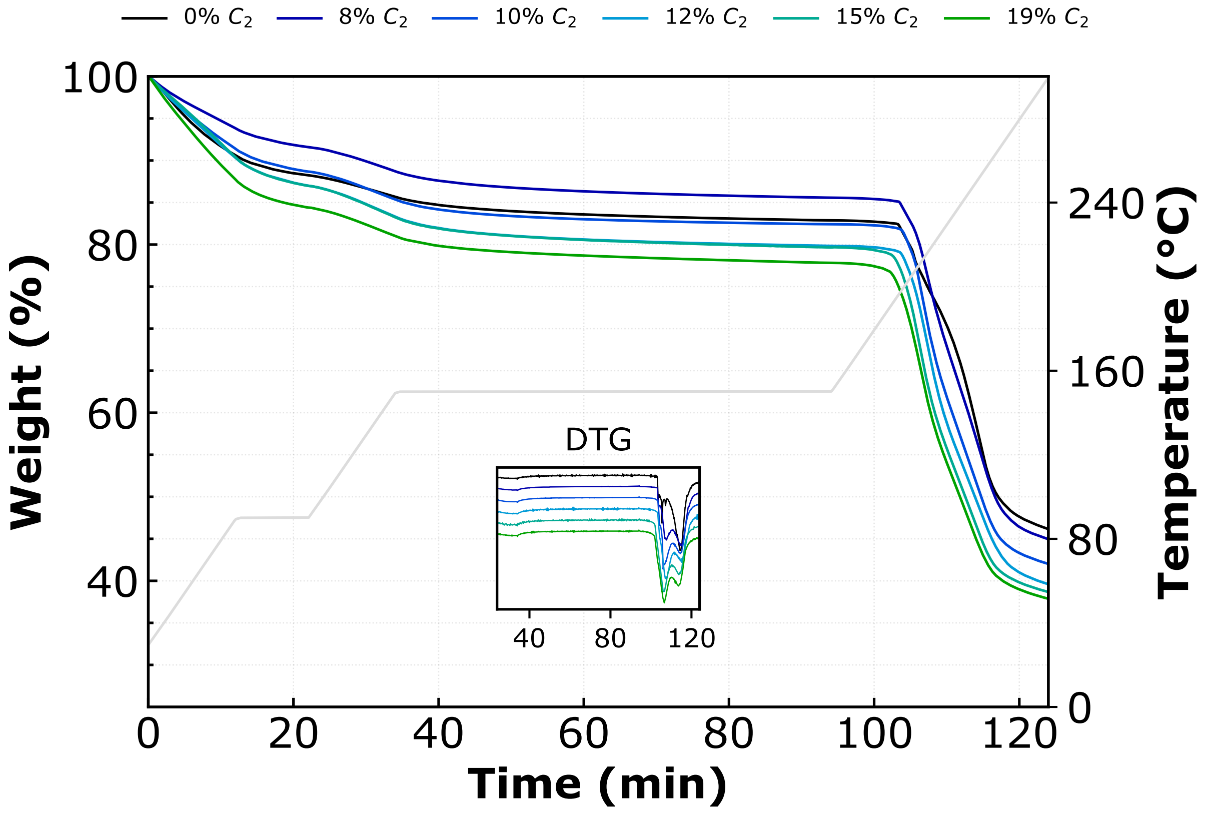

Figure S5. TGA of Na-Alginate-(C_2) films used for determination final plasticizer content via mass loss. Inset plot: Differential Thermogravimetric Analysis (DTG) show mass loss valleys for polyol and alginate.

DMTA of Na-alginate-(polyol) films

DMTA is a very sensitive technique to probe polymer relaxations in a mixture. In the case of blends and plasticized systems, if there is vast different between *Tg(s)*, the individual dynamics can be split into the lower and higher *Tg*. The flexible component (faster relaxation times) will have the lower *Tg* and vice-versa. It is worth mentioning that the lower *Tg* component will have segmental mobility closer to its own bulk. In contrast, the higher Tg one experiences the dynamics of an averaged system[16]. Since the *Tg*(s) estimated from DMTA are a result of changes in segmental mobility on elastic and loss moduli, this technique will also reflect an averaged environment - as in the experienced by the slower or higher component *Tg*. This supports the *Tg* discrepancies normally found between methodologies, e.g., with differential scanning calorimetry. In short, the DMTA analysis will likely show relatively higher *Tg* values and, thus, large positive deviation from the mixture's *Tg* predicted by rules of mixing.

DMTA curves mostly showed one Tg for plasticized Na-alginate materials (Figures S7 to S11). After the Tg event, the rubbery modulus of alginate samples varied with type and size of added sugar-alcohol. Surprisingly, values as high as 1 GPa are obtained for short plasticizers (C_2, C_3) whilst decreased up to 0.01 GPa order of magnitude for C_5 and C_6. It might be that the plasticizer type (size, chirality) influences the degree of semi-crystallinity of alginate blocks under the same casting conditions.

We note that the sample containing C_4 as plasticizer showed signs of separation into two relaxation moduli. The appearance of two Tg-like relaxations is often attributed to partial miscibility of a blend. Furthermore, most samples also show signs of residual water evaporation after the boiling point is reached (appearance of additional secondary relaxations), which is understandable considering hygroscopic samples should still have tightly bound water present.

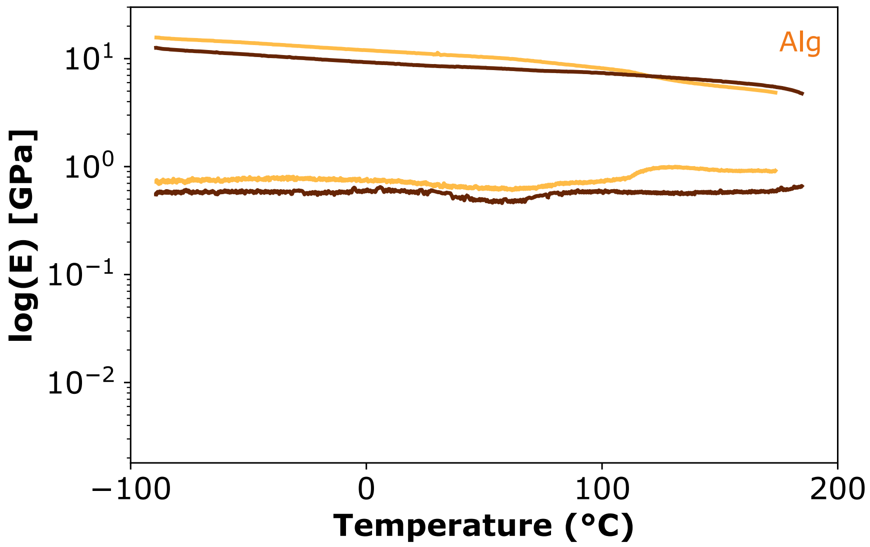

Figure S6. DMTA analysis of dry neat Na-alginate films.

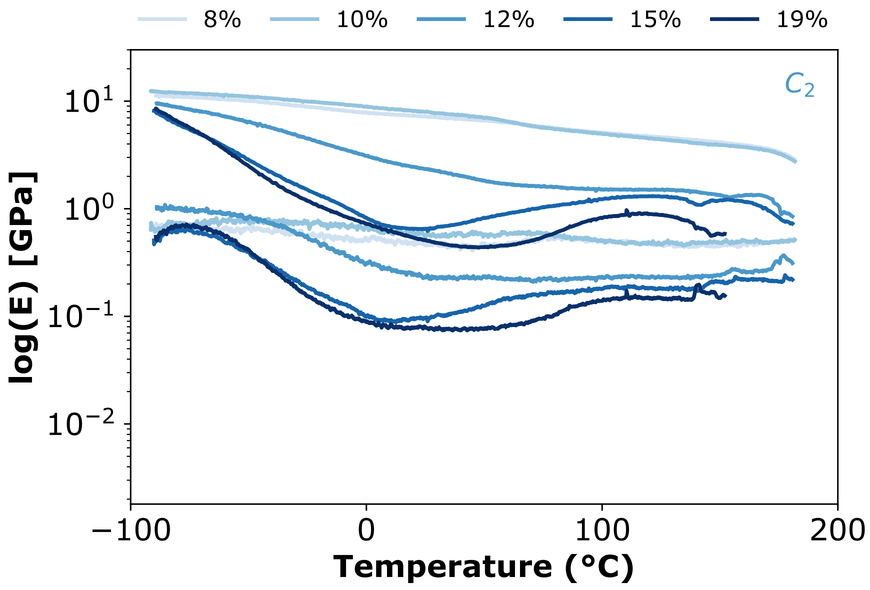

Figure S7. DMTA analysis of dry Na-alginate-(C_2) films.

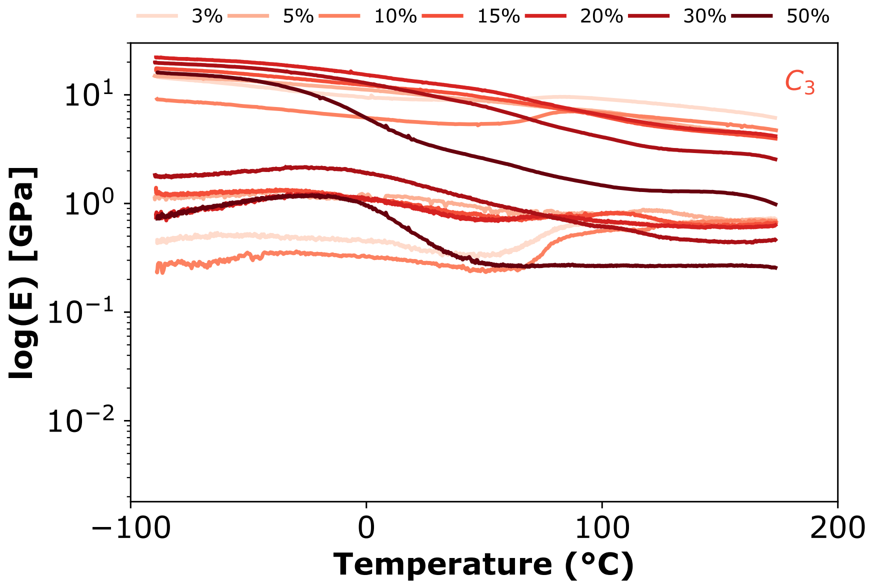

Figure S8. DMTA analysis of dry Na-alginate-(C_3) films.

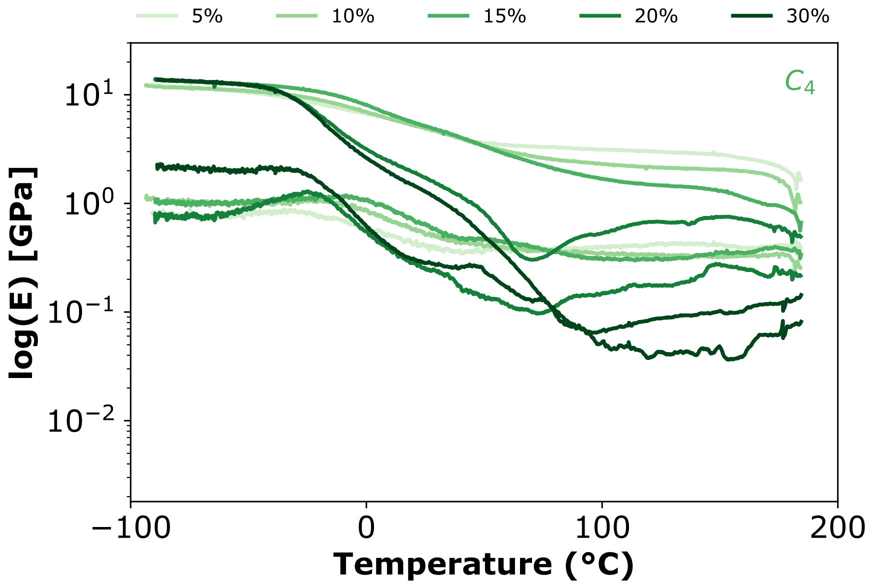

Figure S9. DMTA analysis of dry Na-alginate-(C_4) films.

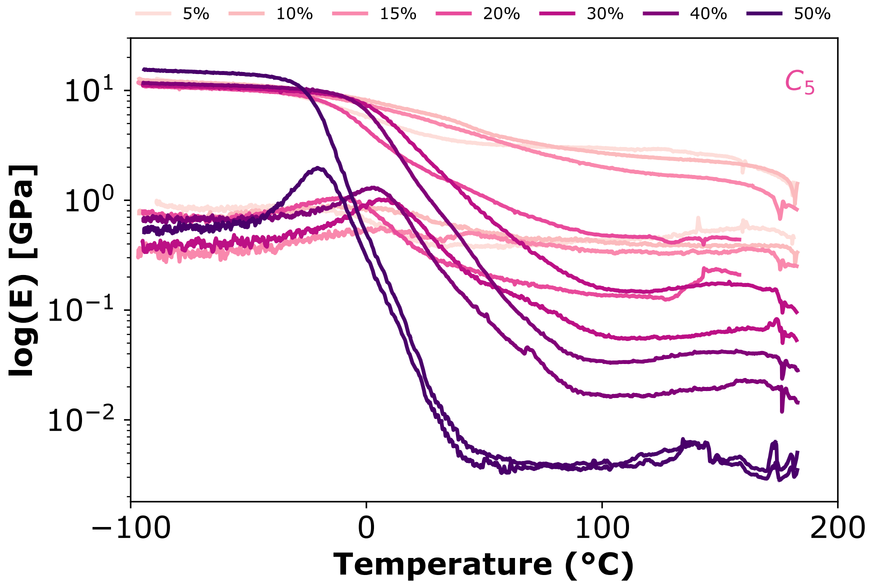

Figure S10. DMTA analysis of dry Na-alginate-(C_5) films.

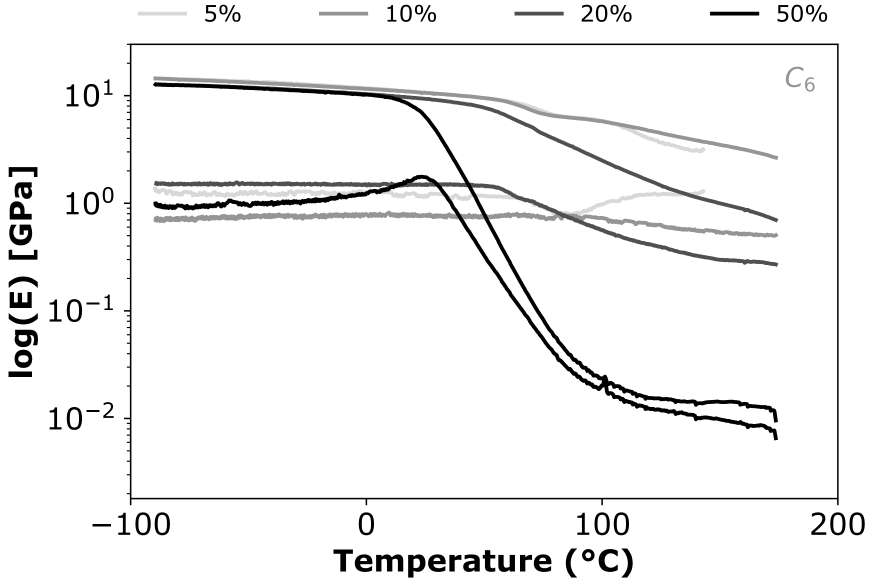

Figure S11. DMTA analysis of dry Na-alginate-(C_6) films.

DMTA and TGA of Na-alginate, C_3, and C_6 polyol films equilibrated to ambient moisture

Water is a natural plasticizer and can be introduced into hygroscopic samples via air humidity. The water content in humidified films, ~50RH, was measured via TGA (Table S1). Plasticized samples with <50 wt.% C_3 or C_6 had similar water content than unplasticized alginate films. The reason for no change in water content with these additions might be the formation of h-bonding between alginate and polyols, leaving no extra sorption sites for water. For high concentration C_3 (50 wt.%), the hydrophilic plasticizer most likely formed concentrated micro-phases that could bind with water (free hydroxyl groups), since the water content was higher than control. For C_6 at high concentration (50 wt.%), the water content was also higher than the unplasticized film, showing water was bound to excess plasticizer. However, this amount was lower than C_3, since it is less hydrophilic. Gao et al. (2017)[17] found similar results for films of alginate-C_3 and alginate-C_6 produced via thermo-mechanical mixing.

As expected, Tg values from DMTA for C_3 and C_6 alginate films varied considerably if under dry or humid conditions (Figure S12). Additionally, we also find relaxation spectra that are close to that composed of two alpha relaxations: one encompassing the plasticizer-rich phase with bound water and yet another for the polymer-rich phase. However, additional investigations would be needed to verify this partitioning, i.e., neutron and x-ray scattering.

Table S1. Water content of Na-alginate films plasticized with C_3 or C_6 sugar alcohols as determined via TGA.

Sugar alcohol	Plasticizer (wt. %)	Water (wt. %)
-	0	18 ± 0
C_3	5	18 ± 0
C_3	20	18 ± 0
C_3	50	36 ± 1
C_6	5	16 ± 0
C_6	20	15 ± 1
C_6	50	27 ± 4

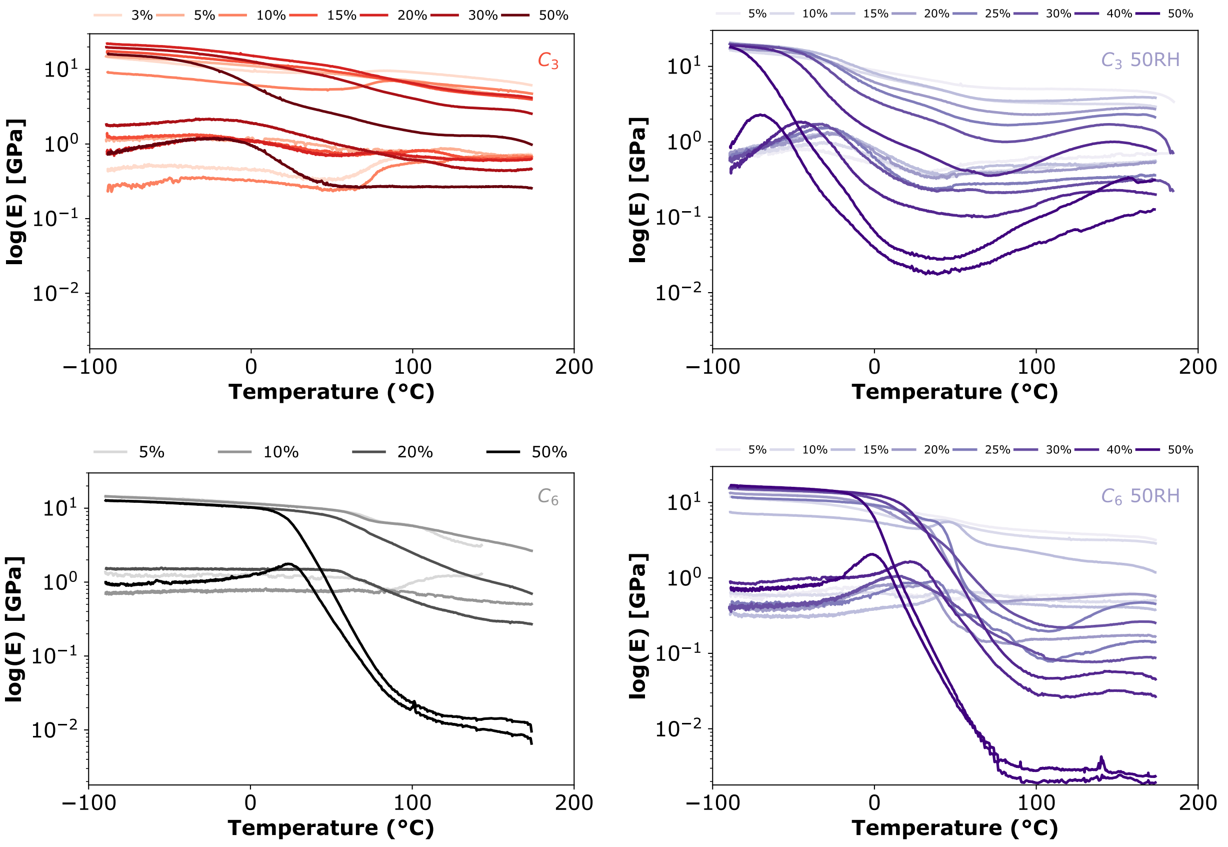

Figure S12. DMTA analysis of Na-alginate-(sugar alcohol) films of glycerol and sorbitol using dry samples (left) and equilibrated to ambient conditions (~50RH, right).

Residuals plot over independent variable for Fox and GML models for alginate-polyols

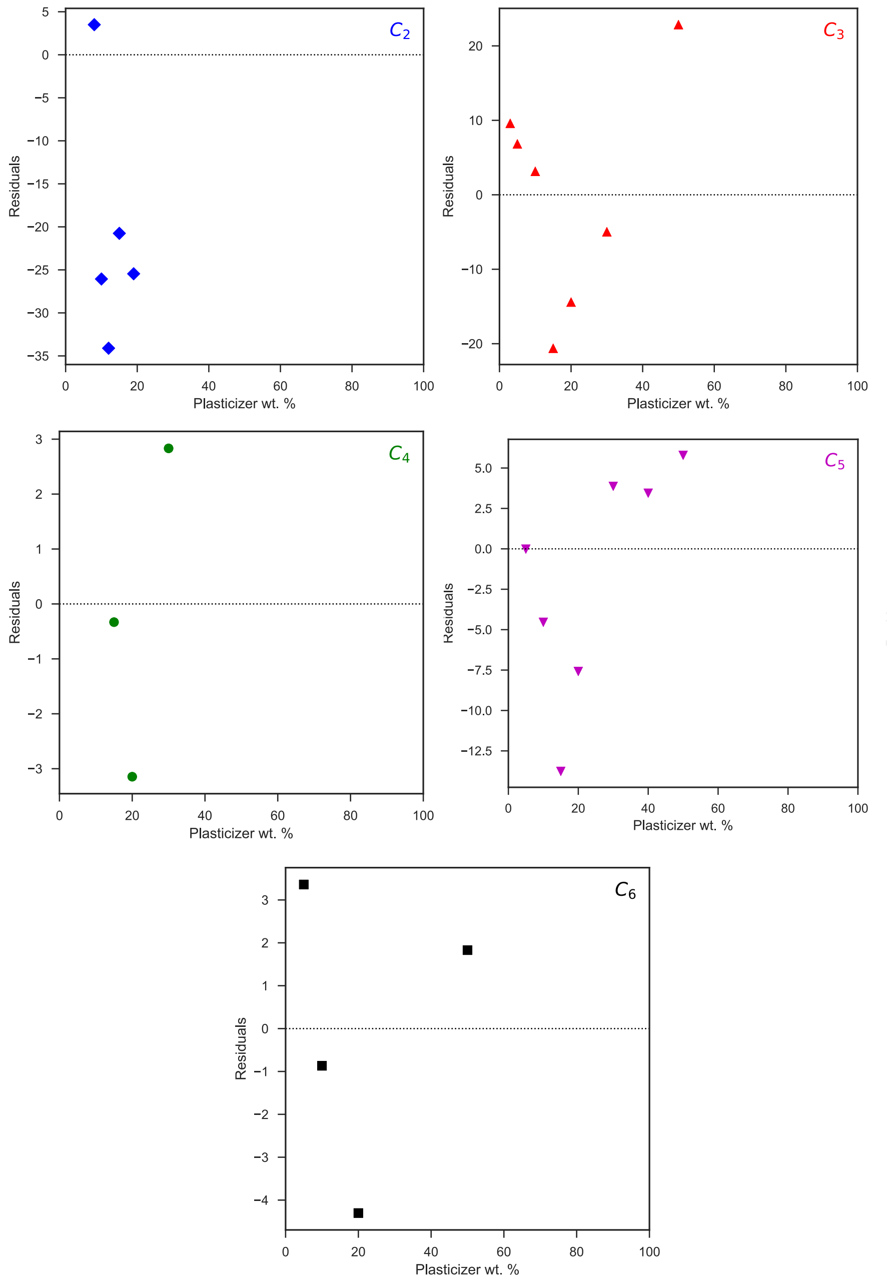

Figure S13. Residuals plot over independent variable (plasticizer wt. %) for the calculated values of glass transition temperature (Tg) using the Fox model for Na-alginate-(sugar alcohol) systems.

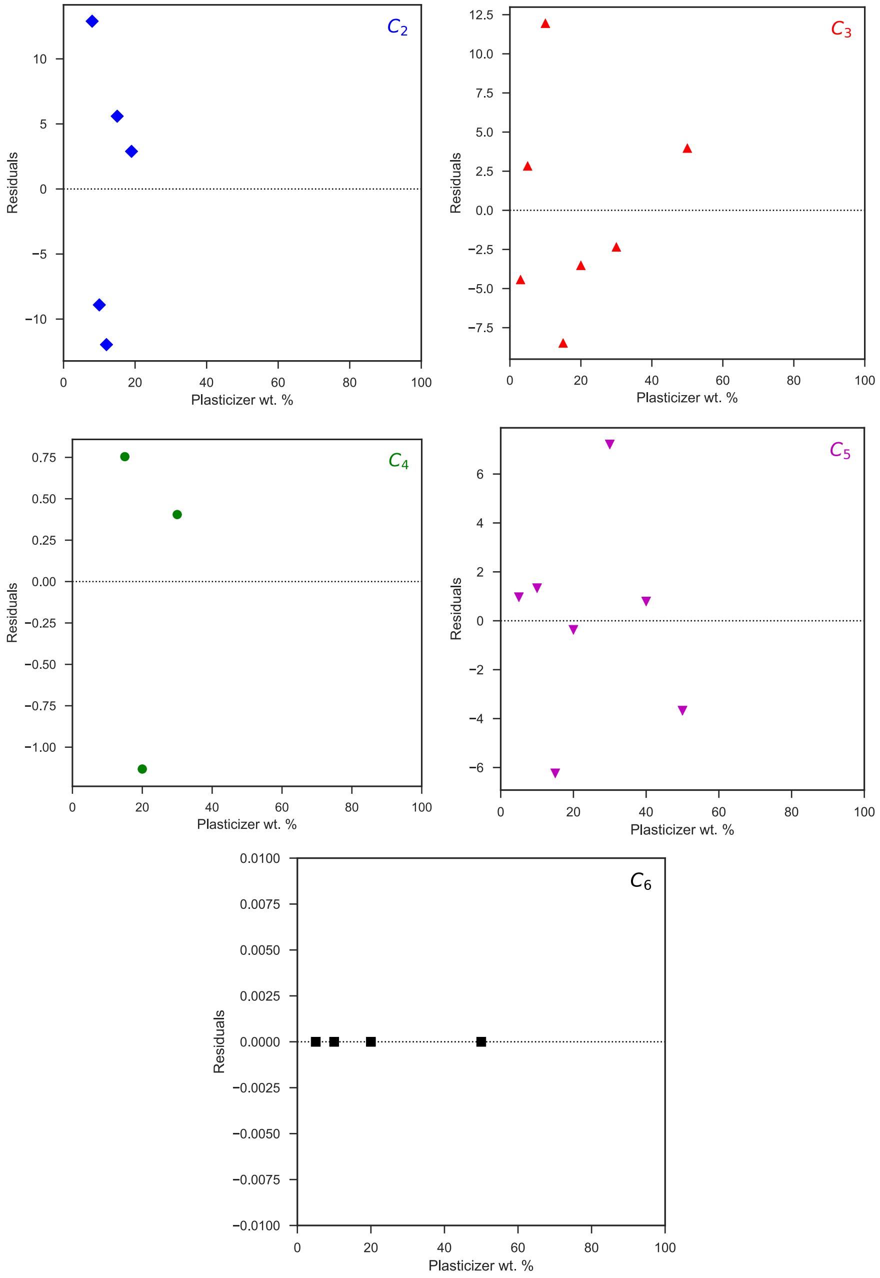

Figure S14. Residuals plot over independent variable (plasticizer wt. %) for calculated values of glass transition temperature (Tg) using the *GML* model for Na-alginate-(sugar alcohol) systems.

Sugar alcohol glass transition temperature

Table S2. Sugar alcohol glass transition temperature from literature and comparison to values from curve fitting Generalised Mean Linear (*GML*) model using a unified *Tg* for the glassy polymer

Polyol	*Literature* *Tg* 2 (°C)	Reference	*Tg* 1 (°C)	Boundaries *Tg* 2 (°C)	*GML* *Tg* 2 (°C)	|*ΔTg* 2| (°C)
C_2	-113	Nagoe and Oguni (2008)[18]	133*	-100 ± 30	-130	17
C_3	-84	Huang et al. (2018)[19]	133*	-79 ± 30	-70	14
C_4	-60	Fujii et al. (2015)[20]	133*	-10 ± 30	-40	20
C_5	-13	Huang et al. (2018)[19]	133*	-22 ± 30	-22	9
C_6	-4	Huang et al. (2018)[19]	133*	-28 ± 30	2	6

* Assumed value for neat Na-alginate as found by Russo and co-workers[4]

|*ΔTg* 2|: absolute difference between literature and curve-fit values of *Tg 2*

Full GM model applied to datasets from literature

The full derivation of the Generalised Mean model, GM, can also be used to fit datasets of systems with higher degree of phase separation. This is accomplished through the exponents alpha and beta, in the expression:

$$Tg = \frac{(\phi_1 {Tg_1}^{\beta} + \phi_2 (Tg_1 k_{GM})^{\beta})^{1/\beta}}{(\phi_1 + \phi_2 (\frac{Tg_1 k_{GM}}{Tg_2})^{\alpha})^{1/\alpha}}$$

where ϕ_i, Tg_i, k_{GM}, α, β are volume or mass fraction of components 1 or 2, glass transition of components 1 or 2, model constant, entropy exponential, and enthalpy exponential, respectively.

In Figure S15, the selected systems from literature have regions of partial miscibility to immiscibility depending on diluent concentration. Therefore, they were fitted with the full GM model (Table S3). Other equations based on Couchman-Karasz theory have been previously adapted to fit such mixtures, with some degree of success. However, our general recommendation is to interpret such systems with the rationale of a phase inversion or plasticizer migration phenomena. Hence, we advise to first do a refined fitting and analysis over the (partially) miscible diluent range, using the linearized GM model. Secondly, analyse the remaining phase-separated system with another GML fitting.

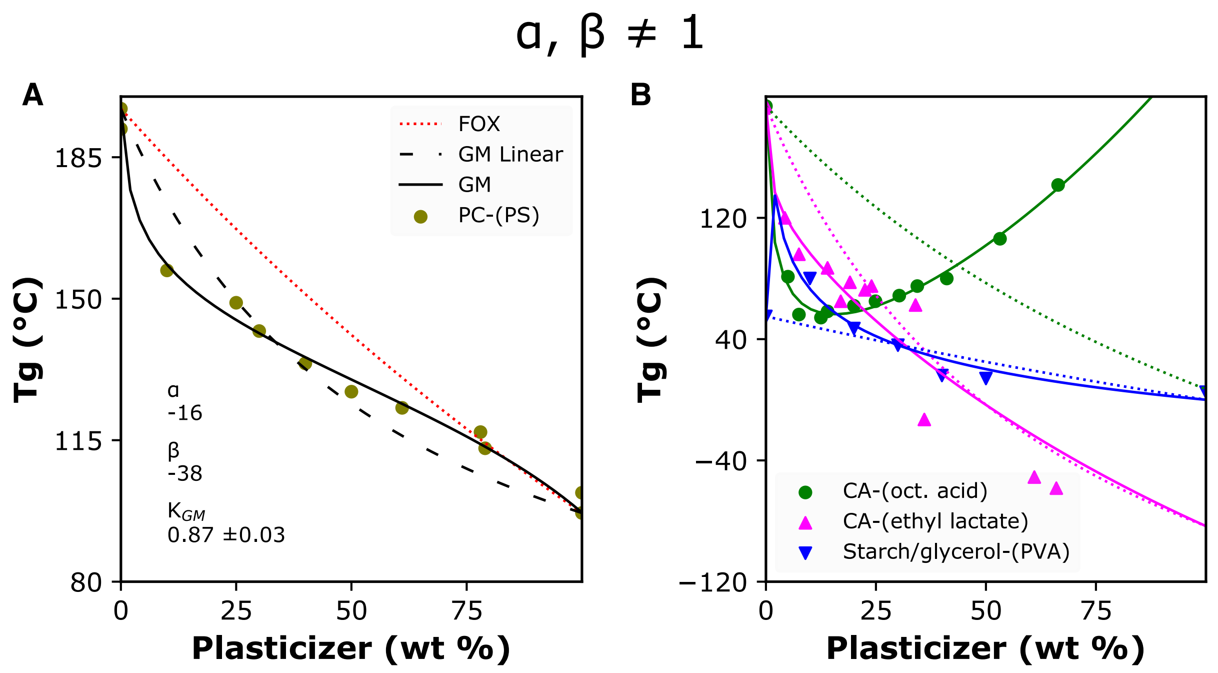

Figure S15. Experimental and calculated values of glass transition temperature (Tg) for several datasets displaying complex deviations from rules of mixing (or α_{GM}, $\beta_{GM} \neq 1$). A) A synthetic polymer blend of Polycarbonate (PC) and Polystyrene (PS). B) Biopolymer-(plasticizer) mixtures of cellulose acetate (CA) and a blend of plasticized Starch/PVA. FOX: Fox model (dotted lines); GML: Generalised Mean Linear model (dot-dashed line); GM: Generalised Mean model (solid lines). For demonstrative purposes, FOX and GML curve-fitting was performed using fixed values for the individual Tg parameters. Data from Schneider 1997[21]; Decroix et al. 2020[8]; Kahvand et al., 2019[22].

Table S3. Glass transition parameters and statistics obtained after curve fitting a Fox model and Generalised Mean models for datasets showing complex immiscibility

System	Tg 1 (°C)	Tg 2 (°C)	Model	Model constant, k (fit ± st. error)	α (fit ± st. error)	β (fit ± st. error)	TSS	P-value	S (°C)	References
PC - (PS)	197	97	Fox	1	1	1	12195	N/A	9.33	Schneider 1997[21]
	197	97	GM	0.87 ± 0.03	-15.94 ± 17.62	-37.59 ± 24.26	11297	3.53E-12	2.87	
CA - (octanoic acid)	194	7*	Fox	1	1	1	41492	N/A	63.14	Decroix et al., 2020[8]
	194	241	GM	2.25 ± 0.21	7.68 ± 2.70	2.94 ± 0.82	19264	2.12E-12	4.19	
CA - (ethyl lactate)	194	-83*	Fox	1	1	1	61010	N/A	32.81	Decroix et al., 2020[8]
	194	-83	GM	0.78 ± 0.15	-1.03 ± 0.82	-30.87 ± 66.74	55949	4.07E-7	20.93	
Starch/glycerol- (PVA)	55	0	Fox	1	1	1	4296	N/A	15.19	Kahvand et al., 2019[22]
	55	0	GM	89.13 ± 264056215.16	3.91 ± 2611662.85	6.54 ± 7315596.32	4271	6.27E-5	6.30	

* Values used to fit models were estimated by this study for illustrative purposes

References

(1) Couchman, P. R.; Karasz, F. E. A Classical Thermodynamic Discussion of the Effect of Composition on Glass-Transition Temperatures. *Macromolecules* **1978**, *11* (1), 117–119. https://doi.org/10.1021/ma60061a021.

(2) Brinke, G. ten; Karasz, F. E.; Ellis, T. S. Depression of Glass Transition Temperatures of Polymer Networks by Diluents. *Macromolecules* **1983**, *16* (2), 244–249. https://doi.org/10.1021/ma00236a017.

(3) Linnenkugel, S.; Paterson, A. H. J.; Huffman, L. M.; Bronlund, J. E. Prediction of the Glass Transition Temperature of Low Molecular Weight Components and Polysaccharide Mixtures. *J Food Eng* **2021**, *292* (June 2020), 110345. https://doi.org/10.1016/j.jfoodeng.2020.110345.

(4) Gordon, M.; Taylor, J. S. Ideal Copolymers and the Second-Order Transitions of Synthetic Rubbers. I. Noncrystalline Copolymers. *Rubber Chem Technol* **1953**, *26* (2), 323–335. https://doi.org/10.5254/1.3539818.

(5) Fox, T. G. Influence of Diluent and of Copolymer Composition on the Glass Temperature of a Polymer System. *Bull. Am. Phys. Soc.* **1956**, *1*, 123.

(6) Guvendiren, M.; McSwain, R. L.; Mates, T. E.; Shull, K. R. Welding Kinetics in a Miscible Blend of High- Tg and Low-T g Polymers. *Macromolecules* **2010**, *43* (7), 3392–3398. https://doi.org/10.1021/ma902679u.

(7) Su, S. Prediction of the Miscibility of Pbat/Pla Blends. *Polymers (Basel)* **2021**, *13* (14), 2339. https://doi.org/10.3390/polym13142339.

(8) Decroix, C.; Chalamet, Y.; Sudre, G.; Caroll, V. Thermo-Mechanical Properties and Blend Behaviour of Cellulose Acetate/Lactates and Acid Systems: Natural-Based Plasticizers. *Carbohydr Polym* **2020**, *237* (February), 116072. https://doi.org/10.1016/j.carbpol.2020.116072.

(9) Kwei, T. K. The Effect of Hydrogen Bonding on the Glass Transition Temperatures of Polymer Mixtures. *J Polym Sci* **1984**, *22* (6), 307–313. https://doi.org/10.1002/pol.1984.130220603.

(10) Brostow, W.; Chiu, R.; Kalogeras, I. M.; Vassilikou-Dova, A. Prediction of Glass Transition Temperatures: Binary Blends and Copolymers. *Mater Lett* **2008**, *62* (17–18), 3152–3155. https://doi.org/10.1016/j.matlet.2008.02.008.

(11) Pinal, R. Entropy of Mixing and the Glass Transition of Amorphous Mixtures. *Entropy* **2008**, *10* (3), 207–223. https://doi.org/10.3390/entropy-e10030207.

(12) Lu, X.; Weiss, R. A. Relationship between the Glass Transition Temperature and the Interaction Parameter of Miscible Binary Polymer Blends. *Macromolecules* **1992**, *25* (12), 3242–3246.

(13) Schneider, H. A. Conformational Entropy Contributions to the Glass Temperature of Blends of Miscible Polymers. *J Res Natl Inst Stand Technol* **1997**, *102* (2), 229. https://doi.org/10.6028/jres.102.018.

(14) Trachenko, K.; Brazhkin, V. v. Heat Capacity at the Glass Transition. *Phys Rev B Condens Matter Mater Phys* **2011**, *83* (1), 014201. https://doi.org/10.1103/PhysRevB.83.014201.

(15) Zetsche, A.; Fischer, E. W. Dielectric Studies of the A-relaxation in Miscible Polymer Blends and Its Relation to Concentration Fluctuations. *Acta Polymerica* **1994**, *45* (3), 168–175. https://doi.org/10.1002/actp.1994.010450306.

(16) Lodge, T. P.; McLeish, T. C. B. Self-Concentrations and Effective Glass Transition Temperatures in Polymer Blends. *Macromolecules* **2000**, *33* (14), 5278–5284. https://doi.org/10.1021/ma9921706.

(17) Gao, C.; Pollet, E.; Avérous, L. Properties of Glycerol-Plasticized Alginate Films Obtained by Thermo-Mechanical Mixing. *Food Hydrocoll* **2017**, *63*, 414–420. https://doi.org/10.1016/j.foodhyd.2016.09.023.

(18) Nagoe, A.; Oguni, M. Glass Transition Behaviors of Ethylene Glycol - Water Solutions Confined within Nano-Pores of Silica Gel. *AIP Conf Proc* **2008**, *982* (February 2008), 185–188. https://doi.org/10.1063/1.2897779.

(19) Huang, C.; Chen, Z.; Gui, Y.; Shi, C.; Zhang, G. G. Z.; Yu, L. Crystal Nucleation Rates in Glass-Forming Molecular Liquids: D-Sorbitol, D-Arabitol, D-Xylitol, and Glycerol. *J. Chem. Phys.* **2018**, *149* (5). https://doi.org/10.1063/1.5042112.

(20) Fujii, K.; Izutsu, K. I.; Kume, M.; Yoshino, T.; Yoshihashi, Y.; Sugano, K.; Terada, K. Physical Characterization of Meso-Erythritol as a Crystalline Bulking Agent for Freeze-Dried Formulations. *Chem Pharm Bull (Tokyo)* **2015**, *63* (5), 311–317. https://doi.org/10.1248/cpb.c14-00692.

(21) Schneider, H. A. Conformational Entropy Contributions to the Glass Temperature of Blends of Miscible Polymers. *J Res Natl Inst Stand Technol* **1997**. https://doi.org/10.6028/jres.102.018.

(22) Kahvand, F.; Fasihi, M. Plasticizing and Anti-Plasticizing Effects of Polyvinyl Alcohol in Blend with Thermoplastic Starch. *Int J Biol Macromol* **2019**, *140*, 775–781. https://doi.org/10.1016/j.ijbiomac.2019.08.185.
